# Porphyrin–Nanocarbon Complexes to Control the Photodegradation of Rhodamine

**DOI:** 10.1021/acsomega.2c05065

**Published:** 2022-11-01

**Authors:** Michael George Spencer, Marco Sacchi, Jeremy Allam, S. R. P. Silva

**Affiliations:** † Quantum Biology Doctoral Training Centre, University of Surrey, GuildfordGU2 7XH, U.K.; ‡ Advanced Technology Institute, University of Surrey, GuildfordGU2 7XH, U.K.; § Department of Chemistry, University of Surrey, GuildfordGU2 7XH, U.K.

## Abstract

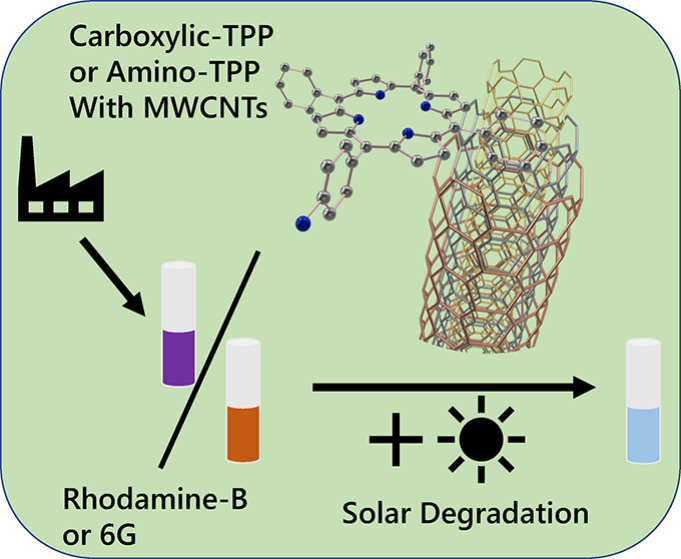

Porphyrin–nanocarbon systems were used to generate a photocatalyst for the control of rhodamine B and rhodamine 6G photodegradation. Carboxylic functionalized multi-walled carbon nanotubes (*o*-MWCNTs) were decorated by two different porphyrin moieties: 5-(4-aminophenyl)-10,15,20-(triphenyl)porphyrin (a-TPP) with an amine linker and 5-(4-carboxyphenyl)-10,15,20-(triphenyl)porphyrin (c-TPP) with a carboxyl linker to the *o*-MWCNT, respectively, with their photocatalyst performances investigated. The optical properties of the mixed nanocomposite materials were investigated to reveal the intrinsic energy levels and mechanisms of degradation. The charge-transfer states of the *o*-MWCNTs were directly correlated with the performance of the complexes as well as the affinity of the porphyrin moiety to the *o*-MWCNT anchor, thus extending our understanding of energy-transfer kinetics in porphyrin–CNT systems. Both a-TPP and c-TPP *o*-MWCNT complexes offered improved photocatalytic performance for both RhB and Rh6G compared to the reference *o*-MWCNTs and both porphyrins in isolated form. The photocatalytic performance improved with higher concentration of *o*-MWCNTs in the complexed sample, indicating the presence of greater numbers of −H/–OH groups necessary to more efficient photodegradation. The large presence of the −H/–OH group in the complexes was expected and was related to the functionalization of the *o*-MWCNTs needed for high porphyrin attachment. However, the photocatalytic efficiency was affected at higher *o*-MWCNT concentrations due to the decomposition of the porphyrins and changes to the size of the CNT agglomerates, thus reducing the surface area of the reactant. These findings demonstrate a system that displays solar-based degradation of rhodamine moieties that are on par, or an improvement to, state-of-the-art organic systems.

## Introduction

Organic pollutants such as the xanthene dyes rhodamine B (RhB) and rhodamine 6G (Rh6G) are a significant source of toxicity in many commercial water streams due to their use in the textile industry and as water flow tracers. Both dyes are thought to contribute to carcinogenic and mutagenic effects on organisms that use such contaminated water streams.^1,2^ As such, efforts are underway to efficiently track and then degrade the dyes into less-toxic products in situ in wastewater environments.

Methods to degrade RhB and Rh6G in aqueous media include the use of photocatalysts and adsorbants—either biological or synthetic—as well as mechanical efforts such as filtration and froth floatation.^3,4^ The use of photocatalysts such as ZnO and TiO_2_ provides a low-cost solution, with increases in efficiency realized with each passing year.^5^ Problems arise with photochemical approaches due to the cost and scalability of efficient photocatalysts as well as the potential for added toxicity of intermediate products.^6,7^ Additionally, the high solubilities of the dyes themselves can lead to low efficiencies of degradation.^8^ The use of solar energy as the light source instead of artificial light sources provides a greener overall degradation solution. For such an application, the use of inorganic semiconductors—with their UV excitation—is not feasible.^9^ Instead, organic semiconductors such as porphyrins and phthalocyanines that can utilize the higher energy end of the visible solar spectrum are of interest.^10^ Further, the low cost and ease of porphyrin spectral tunability as well as the facile synthesis of bound porphyrin–nanocarbon complexes make them an attractive prospect for such studies.^11,12^ Various nanomaterial heterojunction structures have been evaluated for their use as photocatalysts, with both s- and z-scheme heterojunctions showing promise. Examples of this work are shown in papers by Wang et al. and Shen et al.^13,14^

Among the various allotropes of nanocarbons, carbon nanotubes have provided exceptional optical, mechanical, and electrical properties.^15^ Additionally, the attractive chemistry of low-dimensional nanocarbons leads to their widespread use in next-generation devices and structures. The sp^2^ electronic configuration, coupled with the large surface area of nanocarbons, invites many interesting and achievable functionalizations through simple methodologies.^16−18^ Through functionalizations such as carboxylic decoration, samples can overcome inherent carbon nanotube insolubility in water-based solvents.^19−21^

Functionalization of nanotubes also creates environments suitable for a strong binding affinity for chromophore attachment. The use of carbon nanotubes (CNTs) in porphyrin–nanocarbon complexes has a rich history and includes both covalent and non-covalent addition with varying linker lengths.^22−25^

When examining the charge transfer between chromophore–CNT complexes, single-walled carbon nanotubes (SWCNTs) can offer extraordinary and superior transfer properties than multi-walled carbon nanotubes (MWCNTs), including the tunability of energy levels through selective chirality purifications.^26−28^ However, one must look at the cost-effectiveness and large-scale solutions for this problem. The scalability of MWCNT-based complexes is more feasible due to the relative ease of mass production. Overall, the cost of MWCNTs is low compared to such bespoke SWCNT samples.^29^

The tailoring of electrical properties due to defect sites on the carbon nanotube surface must be examined due to the presence of highly defected nanotubes in a sample following acid functionalization to afford the necessary carboxylic groups for chromophore attachment. Defect sites result in changes to electron–hole recombination time that will affect the efficiency of charge transfer and potentially diminish the photocatalytic properties of our sample. However, a recent investigation showed that common defects to the surface of CNTs can increase the recombination times of electron–hole pairs and allow for longer rhodamine–complex interactions to take place.^30^

Porphyrin nanostructures have previously been seen to provide efficient photodegradation of RhB via nanostructurally dependent architectures.^31^ A comparison of the performance of various photodegradation attempts is given in Table 1.

**Table 1:** Comparison of Various Nanocarbon and Porphyrin Photocatalyst Solutions

system comparison				
type	performance	time	illumination (min)	
Ni–GO–CNT	73.6%	UV	120	Hu et al.^6^
Cu_2_O–CuO/TiO_2_	76%	UV	80	Ajmal et al.^9^
porphyrin nanostructure	UV–vis	25%	120	La et al.^10^
porphyrin–GO	22%	UV–vis	120	Larowska^11^
TPP–CNT	16–26%	UV–vis	120	(this work).

In this work, we focus on enhancing the photocatalytic performance of porphyrin–CNT systems by tuning a chemical linker bond between the porphyrin and the CNT anchor. We present evidence indicating a higher bonding affinity, increasing the electron-transfer properties for the complex, resulting in a greater catalytic performance over isolated components of TPP and nanocarbons individually. Additionally, we discuss how a larger *o*-MWCNT concentration leads to further agglomeration over the photoillumination timescale and overall leads to a diminished long-term photocatalytic response.

## Experimental Methods

### Materials

O-MWCNTs in solid form and *N*,*N*-dimethylformamide were bought from Merck (Sigma-Aldrich) and used as prepared. 5-(4-Aminophenyl)-10,15,20-(triphenyl)porphyrin and 5-(4-carboxyphenyl)-10,15,20-(triphenyl)porphyrin were purchased in powder form from Porphychem and used as prepared.

### Synthesis of Porphyrin-*o*-MWCNT Samples

Samples were prepared by weighing and suspending raw o-MWCNTs in DMF to create a high-concentration solution. Initially, 2.7 mg of *o*-MWCNTs were suspended in 13.5 mL of DMF. Additionally, porphyrins were dissolved in DMF to create a high-concentration solution of 2.482 mg in 4.964 mL of DMF. Samples of porphyrins at a fixed loading of 8 μg/mL were prepared, and aliquots were added to diluted solutions of *o*-MWCNTs of various loading concentrations, ranging from 0 μg/mL (reference) to 128 μg/mL, resulting in 4 mL total of solution. The synthesis of the micellar solution preparation follows that of ref (32) in the solution phase, with minor changes through additional tip-sonicating. Briefly, diluted samples were ultrasonicated on 85% power for 1 h in an Ultrawave Qi200 ultrasonic bath, with the temperature of the bath ranging from 23 to 26 °C and the operating frequency between 32 and 38 kHz. Tip sonication was conducted using a 750 watt Cole-Parmer CPX750 at 20% power with a 1 mm tapered tip over 15 minutes using a 3 s on, 5 s off pulse train. Temperatures were monitored, and samples were kept below 20 °C through the use of an ice bath. Horn depth was monitored and kept at 80% solution coverage according to ref (33).

### Optical Characterization

Before each measurement—regardless of the technique—samples were ultrasonicated for 15 min in conditions described above. The UV/visible spectra of samples were recorded using an Agilent Carey 5000 and matched Hellma quartz cuvettes with a 10 mm path length at room temperature. Steady-state photoluminescence spectra were collected on a PicoQuant FluoTime 300 spectrometer, with emission captured and f-matched with the monochromator through a 2 in. lens; this was passed through a 450 nm edge pass filter and spectrally resolved through a double monochromator using additive gratings. The detector used was a PMA-hybrid -07 detector with a 6 mm active area. Emission was measured with a 403.9 nm excitation with a 1.3 nm spectral width at a 371.2 mW power, with a 1.2 mm spot size. Calibration curves were provided and applied throughout. Measurements were taken in solution at room temperature with cuvettes of 10 mm path length. Time-resolved PL spectra with acquisition times from 0 to 100 ns were taken on a PicoQuant FluoTime 300 spectrometer, typically with a 20 ps IRF using a diluted LUDOX solution as purchased from a PicoQuant. A calibrated PMA-hybrid -07 detector is used, with 6 mm active area. Solutions were transferred to matched Hellma quartz cuvettes for measurements. Emission was measured with a 403.9 nm excitation with a 1.3 nm spectral width at a 371.2 mW power, with a 1.2 mm spot size.

### Photodegradation Experiments

#### Photocatalyst Performance

Illumination experiments to determine the photocatalytic properties of our samples with respect to the degradation of rhodamine moieties were carried out in solutions of DI water at pH 7 at ambient temperature. After sample synthesis, the mixture was stirred in the dark until it reached an adsorption–desorption equilibrium. Following this, an aliquot of sample was taken from a top-down approach of 300 μL of the mixed solutions to give the pre-illumination absorption response. Measurements were conducted with clean quartz cuvettes. The sample was continuously irradiated using a Class-B spectral match solar simulator (25 W/cm^2^ with a cut-off filter λ > 400 nm). The decrease of the rhodamine moiety concentration was measured spectrophotometrically at 554 and 522 nm for RhB and Rh6G, respectively. These measurements were repeated three times for each concentration sample. The spectrum of the solar simulator, and match to the measured solar spectra, is shown in the Supporting Information in Figure S31.

## Results and Discussion

### Steady-State Absorption Measurements

**Figure 1:**
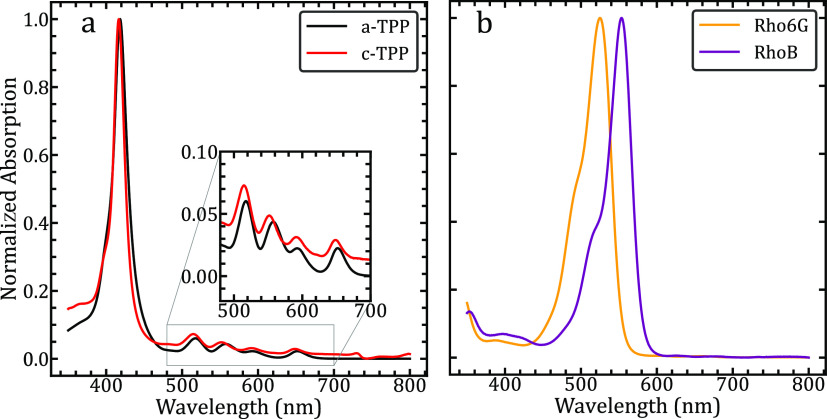
(a) Normalized steady-state absorption spectra of both a-TPP and c-TPP show a strong Soret band with a higher energy shoulder near 420 nm. The inset figure displays the four vibrational Q-bands highlighting the symmetry of the center of the porphyrin molecule. (b) Normalized steady-state absorption spectra of the toxic dyes Rh6G and RhB, highlighting the difference in absorption maxima. Each molecule displays a strong high-energy shoulder of the main absorption peak.

The free-base porphyrins used in this paper each displayed a typical absorption response in DI water, with the presence of a strong Soret band at 416 nm for a-TPP and 418 nm for c-TPP as shown in Figure 1A. Additionally, four vibrational (Q) bands were seen for both a-TPP and c-TPP that were situated between 500 and 700 nm for both porphyrin isolates. This response follows predictions of the four-orbital model by Gouterman.^34^ A typical absorption response for the *o*-MWCNTs can be seen in Figure S1, with an ensemble of states forming a near continuum of absorption response in the region measured from 350 to 800 nm. There is a noticeably higher response at the higher energy near-UV wavelengths.

In Figure 1B, we have shown the absorption response in DI water for the rhodamine dyes of interest: RhB and Rh6G. Maxima were observed for Rhb at 550 nm and Rh6G at 520 nm, with a strong response and a higher energy shoulder.

When creating the porphyrin–nanocarbon complexes, the steady-state absorption response for the a-TPP–*o*-MWCNT and c-TPP–*o*-MWCNT complexes increases linearly over the concentrations tested. Additionally, because of the preference for bonding via the A3B-linker molecule of the porphyrin to the functionalized surface of the CNT, we do not expect a substantial change in the curvature of the porphyrin molecule from a π–π bond that itself would lead to a shift in absorption maxima.^35^ To assess the affinity of porphyrin adsorption onto the nanotube surface, we filtered the porphyrin–CNTs out from the sample, initially by centrifuging the samples and then by filtering the re-dispersed precipitate pellet. The supernatant itself was tested for the presence of CNT as well to confirm a separation of free porphyrins and those adsorbed to the CNT structure.

## Steady-State Fluorescence Measurements

To assess the electronic interaction between the adsorbed porphyrin and the CNT scaffold, fluorescence measurements at different concentrations of CNTs were conducted. Given the metallic nature of MWCNTs, higher concentrations of *o*-MWCNTs resulted in a substantial decrease in fluorescence yield due to the fast and efficient luminescence quenching of the porphyrin excitation.^28^ Such a feature arises due to the mixing of intra-tube density of states within the individual MWCNT structures.^36,37^

**Figure 2:**
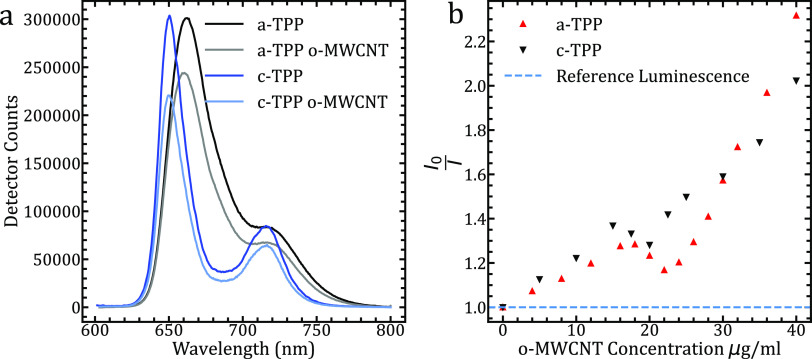
(a) Normalized steady-state fluorescence spectra of a-TPP and c-TPP in isolates and when affixed onto *o*-MWCNTs. Two peaks are observed for each porphyrin, with c-TPP emitting at higher energies. (b) Stern–Volmer plot displaying the photoluminescence quenching of both a-TPP and c-TPP by the acid-functionalized *o*-MWCNTS. We observed a decrease in luminosity between the *o*-MWCNT concentrations of 18 and 25 μg/mL. There is a larger quenching by c-TPP at *o*-MWCNT concentrations below 30 μg/mL, and this trend is reversed at higher concentrations.

Typical steady-state fluorescence spectra of a-TPP and c-TPP can be seen in Figure 2A. We observe two main peaks representing the (0, 0) ground-state → ground-state and (0, 1)-band ground-state → first vibrational state, respectively. Here, it is observed that c-TPP has higher energy fluorescence emission and a sharper peak shape. Upon the addition of CNTs to the TPP sample and following complex formation, some luminescence is quenched due to energy and charge transfer between the TPPs and CNTs when excited in the Soret band.^38^ However, not all luminescence is quenched due to an observed resonant effect ascribed to the porphyrin–porphyrin interaction on the complex surface.^29^ Additionally, some free porphyrins will be present in the solution due to photocleaving from the complex surface and due to an imperfect filtration process during complex synthesis.

The decrease in fluorescence intensity indicates a strong interaction between the porphyrin and CNT surface as predicted by similar studies using both covalent and non-covalent adsorption of porphyrin molecules onto MWCNT surfaces. As observed previously, the quenching at MWCNT concentrations ranges up to 50 μg/mL with a fixed concentration of TPP at 8 μg/mL, leading to a non-linear Stern–Volmer response, as can be seen in Figure 2B.^29^

When combined with minimal changes to the absorption spectra, this would indicate the presence of an additional dynamic quenching mechanism. Due to the substantial coverage of the highly defected o-MWCNT by the porphyrin complexes, the non-linear quenching was prescribed to the porphyrin–porphyrin interaction. Briefly, the average interaction distance existed at the limits between the Dexter to the Förster transfer regime at these concentrations due to trends in o-MWCNT agglomerate sizes. Additional reverse-saturable and saturable absorber effects of the porphyrin–CNT complexes lead to this unusual luminescence quenching behavior.^39^

## Time-Resolved Fluorescence Measurements

**Figure 3:**
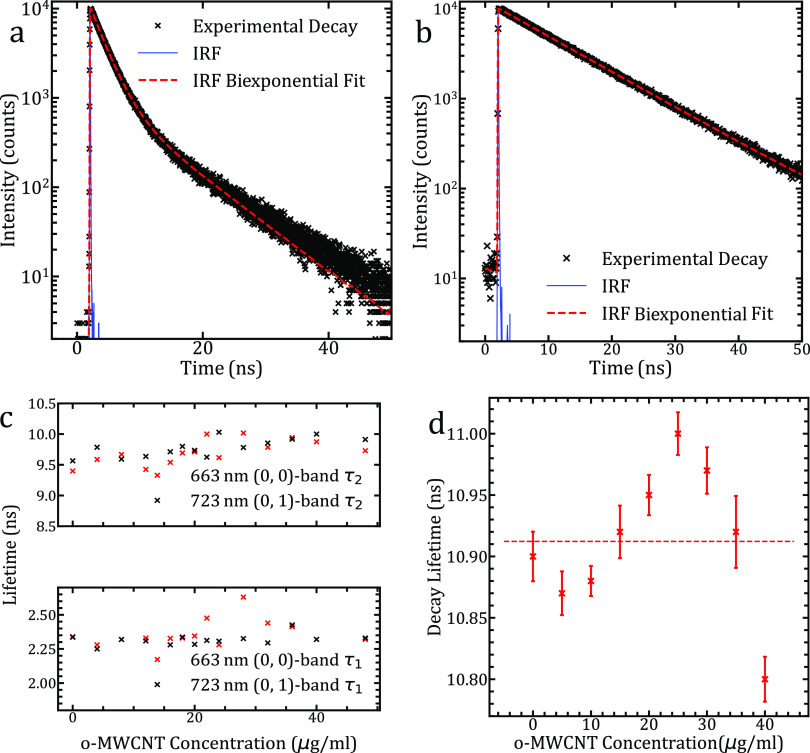
(a) Biexponential decay spectra of the (0, 0) band of a-TPP at 663 nm, using λ_exc_ = 405 nm, showing a biexponential fit reconvoluted with the IRF. (b) Biexponential decay spectra of the (0, 0) band of c-TPP at 663 nm, using λ_exc_ = 405 nm, showing a biexponential fit reconvoluted with the IRF. (c) Lifetimes of both components of the (0, 0) and (0, 1) bands of a-TPP plotted against concentration. There are only slight variations in the lifetime of the components across the range tested. (d) Lifetimes of the (0, 0) and (0, 1) decay of c-TPP plotted against concentration. A single component of the decay is observed rather than the biexponential of a-TPP. While the changes are small over the concentration, a peak in the length of decay lifetime occurs at the peak of the resonant concentration for non-linear quenching behavior.

Under direct excitation of the Soret band, the relaxation mechanisms of the porphyrin excitation can be observed. Despite much of the excitation being quenched by the *o*-MWCNTs, significant statistics can be gained for singlet decay of both a-TPP and c-TPP. An example decay of a-TPP is shown in Figure 3A.

It is clear that there are two components to the lifetimes of the 663 nm peak of a-TPP: 2.35 and 9.5 ns with the faster decay being stronger for the isolated porphyrin, and nearing equal contributions in the complex. Here, a biexponential decay model indicates that a charge separation state exists within the porphyrin–nanocarbon system.^40^ The decay lifetimes of both 663 and 753 nm peaks are taken at the magic angle and are plotted with respect to concentration in Figure 3C. We can see from this data that the lifetimes of the two components for each peak are mainly unchanged across the concentrations of the samples measured.

For c-TPP–*o*-MWCNT complexes, we see the emergence of a single-lifetime decay, as highlighted by the straight line of the log plot in Figure 3B. A longer decay of almost 11 ns in the singlet state is observed that does vary minimally (sub-2% of the lifetime) across the concentration. However, the trend across concentrations has a defined peak around the concentration of maximal non-linear quenching behavior as can be seen in Figure 3D. Despite the minor difference between the porphyrin moieties, namely, the A3B functional group, we observe a vastly different time-resolved PL response. We conclude that the functional group difference does significantly alter the coupling of the TPP to the *o*-MWCNT surface. Expected bond lengths and structure of the morphologies would also be expected to be markedly different.

## Photodegradation of RhB

Our two complexes were individually utilized as the photocatalyst for visible light photodegradation of RhB over short illumination times of up to 2 h. The effect of *o*-MWCNT concentration was probed to assess the efficiency of photodegradation and the longevity of the sample to continued degradation over longer time periods. Porphyrin–nanocarbon complexes in DI water were ultrasonicated for full dispersion prior to the addition to rhodamine mixtures, also in DI water. Following this, the samples were ultrasonicated in the dark and then stirred in the dark continuously for 4 hrs as well as continually stirred over the course of the illumination. To monitor degradation, the RhB peak at 550 nm was tracked through absorption measurements. The absorption measurement itself was investigated for degradation itself, with data presented in Figure S2, and considered negligible for the time and intensity of the measurement.

**Figure 4:**
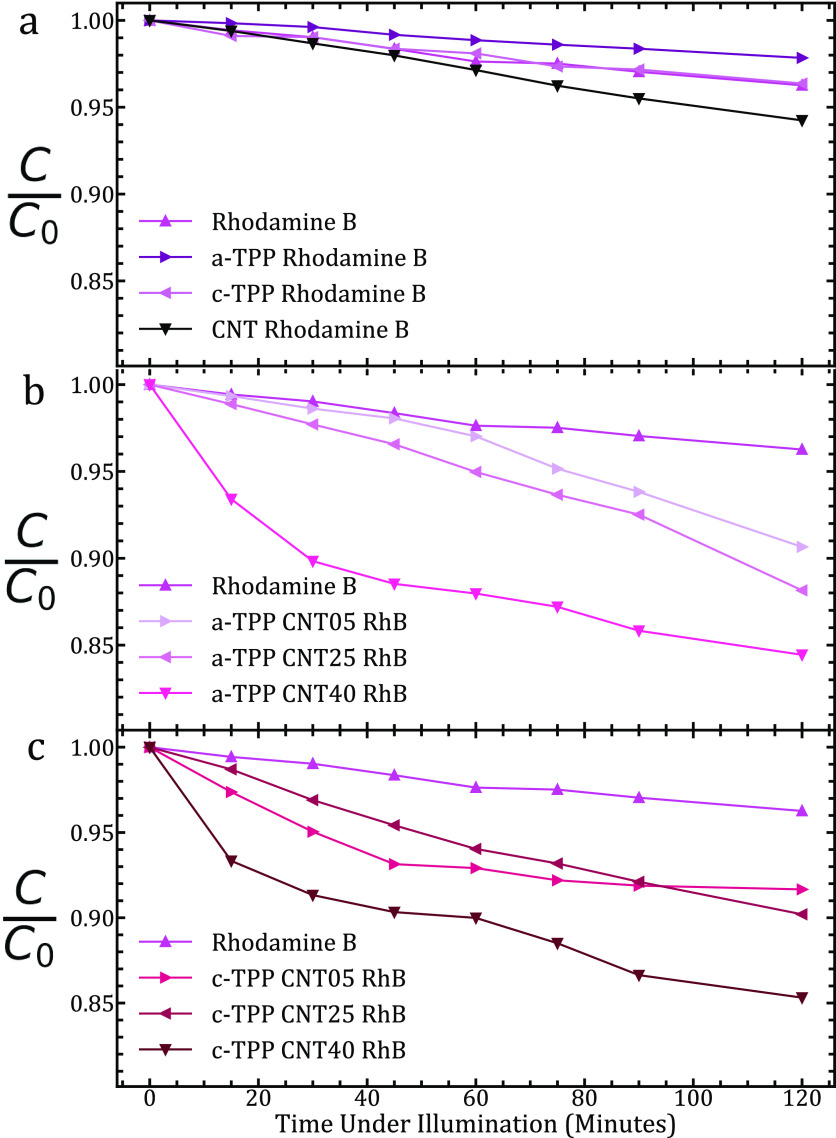
(a) Degradation of RhB under photoillumination with just the control sample, and the addition of a single component of the TPP–MWCNT complexes to assess their individual photocatalytic effect. (b) Photodegradation of RhB following the addition of a-TPP–MWCNT complexes. Observe the change from a linear degradation with time at lower CNT concentrations to a near-exponential relationship at 40 μg/mL, indicating a change within the sample over the course of the experiment attributed to CNT agglomeration. (c) Degradation of RhB under photoillumination following the addition of c-TPP–MWCNT complexes. Observe the change from a linear degradation with time at lower CNT concentrations to a near-exponential relationship at 25 and 40 μg/mL, indicating a change within the sample over the course of the experiment attributed to CNT agglomeration.

Control experiments were conducted to assess the degradation of RhB in DI water without the presence of other species, as well as RhB with the individual components of the complexes: *o*-MWCNTs and both free a-TPP and c-TPP isolates. Some degradation is observed and is shown in Figure 4A, but all datasets are within 6% degradation over the course of the 2 h of illumination. This data shows that the porphyrins have a small effect to the control RhB degradation, with a slight upturn in degradation caused by the addition of *o*-MWCNTs. The unusual reduction of the photodegradation response of the rhodamine when a-TPP moieties are added is assigned to an increased stabilization of the solution. The Soret response in the absorption spectra, as can be seen in Supporting Information Figures S5 and S6, increases over the photoillumination timescales. This response indicates some complexing and stable aggregation of the rhodamines to the a-TPP porphyrins that will reduce sensitization across the experiment.

The performance of the varying CNT concentrations and two porphyrins in their complexes are shown in Figure 4A,B. We can see how the photocatalytic performance increases with *o*-MWCNT concentration. This is due to a higher level of porphyrin attachment to the CNT scaffold. There is a change between a linear relationship of degradation with time to a near-exponential curve with increasing CNT concentration, indicating a change within the sample over the course of the experiment. This is attributed to CNT agglomeration over the experimental photoillumination time, which reduces the surface area for porphyrin–rhodamine interactions to occur. This exponential line shape is not seen at *o*-MWCNT concentrations of 5 μg/mL of any TPP sample. A comparison of the performance of UV and UV–vis systems to our complexes is given in Table 1. We can see that UV–vis photocatalysts have a generally lower performance but have the advantage of solar illumination as the excitation source. Our system is on par with other UV–vis photocatalysts.

Photodecomposition of porphyrins was also monitored, and this determines the longevity of a sample to be useful as a photocatalyst for photodegradation of other compounds. Decomposition within the absorption spectra is shown in Figures S4–S26 (even numbers) and tracked for peak height in Figures S5–S27 (odd numbers). We observe how the addition of CNTs at high concentrations of 40 μg/mL introduces porphyrin decomposition similar to observed rhodamine degradation under photoillumination, whereas a MWCNT concentration of 5 μg/mL has minimal decomposition.

The linear degradation of the control experiments and the lower CNT concentrations of the porphyrin–nanocarbon complexes transitions to an exponential trend at the higher CNT concentrations. This exponential line shape would suggest a reduction in photodegradation performance over a longer time period for the higher concentrations compared the longevity offered but lower efficiency of the lower CNT concentrations. We attribute this to the formation of larger CNT agglomerates triggered by the extra thermal energy of the lamp impinging the RhB solutions.^41^ Larger agglomerates would lower the surface area available for RhB–porphyrin complex interaction.

**Figure 5:**
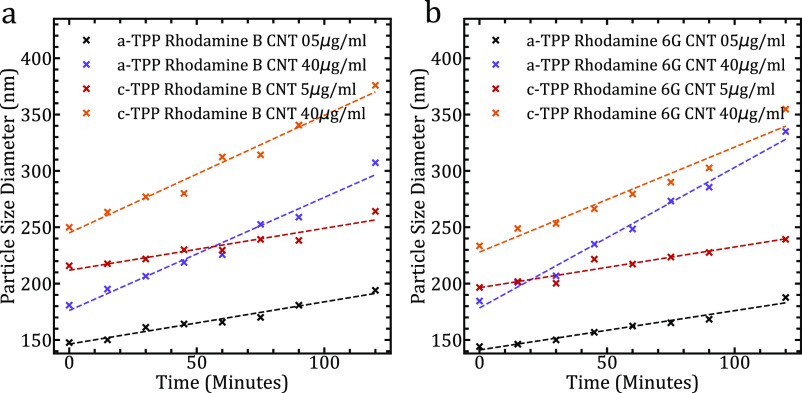
(a) Dynamic light scattering data comparing particle size for a-TPP–*o*-MWCNT complexes with those of c-TPP–*o*-MWCNTs in RhB DI–water solutions over the 2 h photoillumination experiment. (b) Particle size data of a-TPP–*o*-MWCNT complexes and c-TPP–*o*-MWCNTs with Rh6G in DI–water solutions. A linear fit for each trend is applied.

To evaluate the effects of particle size in solution, we took dynamic light scattering data from our samples. The response for RhB is shown in Figure 5A. Focusing on the larger agglomerate making up the bulk of the DLS distribution, we observe an increase in agglomerate size over the illumination time of the experiment. In general, c-TPP–*o*-MWCNT agglomerates were 50 nm larger than those complexed with a-TPP. Additionally, the gradient of increase in agglomerate size over the 2 h period greatly increases with MWCNT concentration. A linear relationship can be fitted to the higher concentration, although a slight exponential bowing is observed that may account for the increasingly exponential line shape of the degradation for the 40 mg/mL samples. Additional rhodamine reference solution particle size data is given in the Supporting Information.

The photodegradation of RhB occurs via N-deethylation and requires reactive oxygen species as provided following the photoexcitation of organic photocatalysts and the carboxylic functionalized MWCNT as well as the DI water solvent environment.

**Figure 6:**
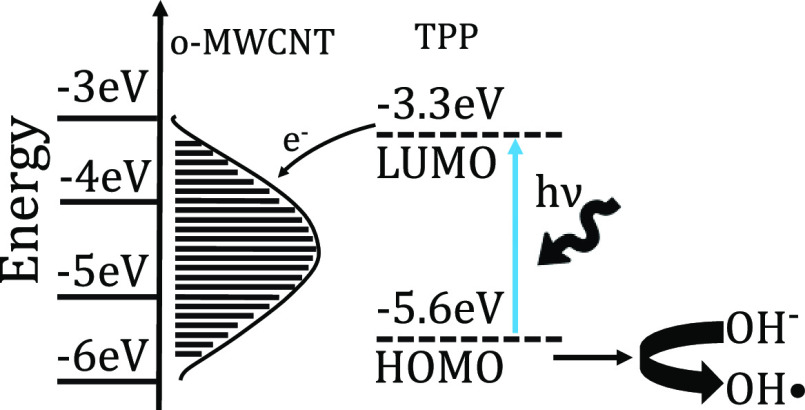
Energy-level diagram for the excitation of porphyrin–CNT complexes, subsequent electron transfer, and hydroxyl radical formation that leads to photocatalysis of rhodamine moieties.

Upon photoexcitation, the porphyrin molecule enters the *S*_1_^*^ LUMO level and transfers an electron efficiently to the *o*-MWCNT surface, with a work function in the range of 4.6–5.1 eV, leaving a photogenerated hole.^42−44^ This hole can react with H_2_O to form a highly reactive hydroxyl radical OH^•^ that can oxidize RhB to create degraded products.^11^ The hydroxyl radical is formed by the reaction of the electron hole left within the porphyrin–CNT complex and the solvent. This is a common scenario when recombination rates are low, as is the case within our complexes.^45,46^ This is explored within Figure 6. Expected HOMO/LUMO levels are taken from ref (46).

Adsorption of rhodamines to the CNT surface is unlikely when we consider the harsh high-energy synthesis methods required to create the porphyrin–CNT complexes. This synthesis—involving temperature tip sonication and ultrasonication while stirring—is not conducted after the addition of the rhodamines. As such, we expect little adsorption of rhodamines to decorated CNT surfaces.

Additionally, the photogenerated electron transferred to the MWCNT can produce the oxygen superoxide anion O_2_^•–^ that can react with the present hydrogen peroxide and create further hydroxyl radicals to participate in photodegradation according to eqs 1–6.
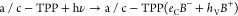
1
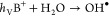
2
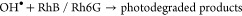
3
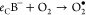
4
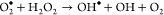
5
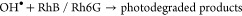
6

The presence of singlet oxygen that could be utilized for photodegradation properties is expected to be minimal due to the suppression (1/1000 of singlet) of the triplet states of our a-TPP porphyrin–nanocarbon complexes as can be seen in Figure S3.^47^ No triplet states were observed for complexes synthesized with c-TPP.

## Photodegradation of Rh6G

Identical experiments to those conducted to photodegrade RhB were conducted to see the performance of the photocatalysts to degrade Rh6G. As such, experimental parameters including rhodamine concentrations and those for the photocatalysts were unchanged to allow for a direct comparison.

**Figure 7:**
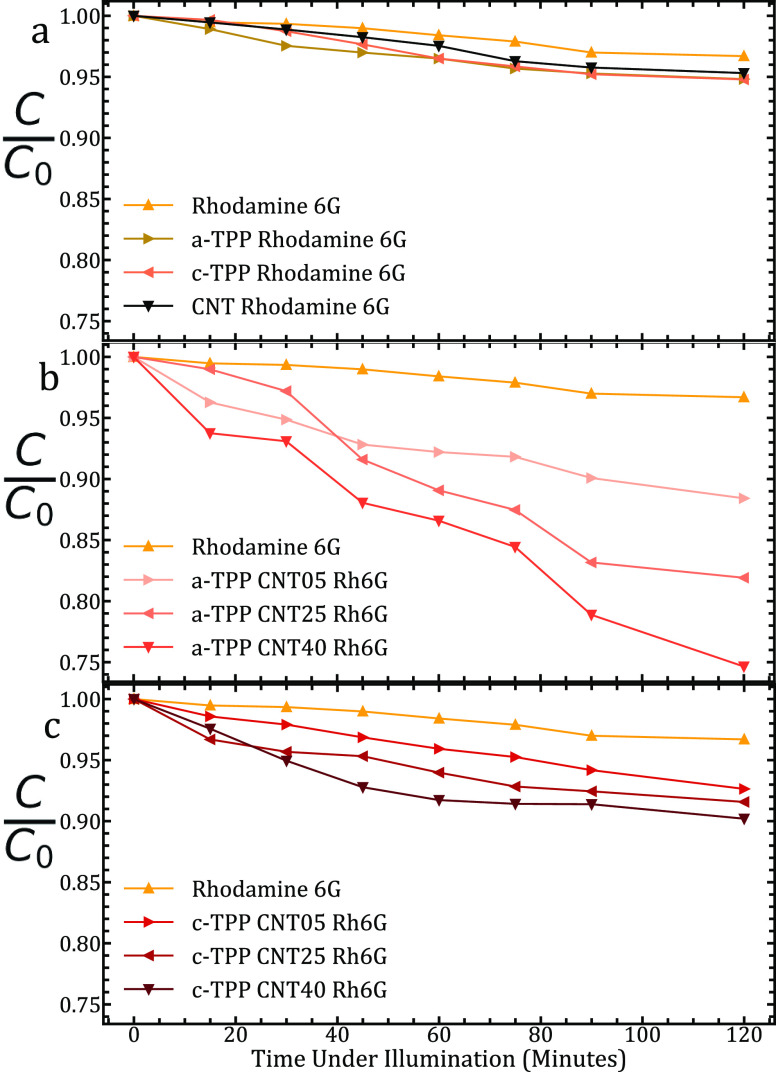
(a) Degradation of Rh6G under photoillumination with just the control sample and the addition of a single component of the TPP–MWCNT complexes to assess their individual photocatalytic effect. (b) Photodegradation of Rh6G following the addition of a-TPP–MWCNT complexes. The CNT concentration per complex is varying. Observe the change from a linear degradation with time at lower CNT concentrations to a near-exponential relationship at 40 μg/mL, indicating a change within the sample over the course of the experiment attributed to CNT agglomeration. (c) Photodegradation of Rh6G following the addition of c-TPP–MWCNT complexes. Observe the change from a linear degradation with time at lower CNT concentrations to a near-exponential relationship at 25 and 40 μg/mL, indicating a change within the sample over the course of the experiment attributed to CNT agglomeration.

Starting with reference data, we investigated the degradation of Rh6G under lamp illumination for the 2 h duration of typical experiments. The data can be seen in Figure 7A. Over the 2 h, the reference sample degraded by just over 3%, displaying some interaction with the light on its own and indicating energetic favorability in the degraded state. Upon the addition of the porphyrins individually, an increase in degradation to over 5% following 2 h is observed. This is in contrast to the sub-5% degradation when just the *o*-MWCNTs are added to the rhodamine sample. This is in contrast to the reference data for RhB in which the CNT sample displayed the greatest degradation. Throughout these reference curves, a distinct flattening is observed at the latter time steps, indicating some decomposition of the photocatalysts and an expected decrease in photocatalytic activity beyond the 2 h mark.

To observe the effect of the porphyrin–nanocarbon complexes as photocatalysts, we again added pre-synthesized a-TPP–*o*-MWCNT and c-TPP–*o*-MWCNT samples with three different CNT concentrations to new rhodamine solutions before illumination. After ultrasonication and stirring in increments discussed previously, the samples were illuminated, and the rhodamine response was monitored over a 2 h time window for photocatalytic activity.

From the response of a-TPP–*o*-MWCNTs as can be seen in Figure 7B, we observe a vast improvement in catalytic activity for all complexes in comparison to the reference data. In general, the performance improved upon the addition of more CNTs to the complex sample with a fixed porphyrin concentration. In the samples, the porphyrin is excited and acts as the electron donor. Rates of electron transfer in the samples increase with MWCNT concentration, with an excited state able to easily find an electron acceptor in the form of the metallic MWCNT structure. We also observe a linear trend of catalytic performance for all the three samples. Overall, the performance for a-TPP–*o*-MWCNT complexes ranges from 10% for the 5 μg/mL sample to over 25% for the 40 μg/mL sample. This represents the largest degradation seen in this work.

The addition of c-TPP–*o*-MWCNT complexes to a fresh rhodamine-6G solution offered contrasting results to the aminophenyl-linked porphyrin. General trends of increasing CNT concentration as can be seen in Figure 7C led to a rise in photocatalytic response but at a lower magnitude. Although the catalytic performance was improved compared to the use of individual components, even at the highest CNT level, we observe a performance over 2 h of less than 10%. We also observe an exponential shape to the highest CNT concentration sample (40 μg/mL), indicating a diminishing performance of the photocatalyst environment over the 2 h of illumination across the experiment.

Again, we evaluate the DLS particle size data as shown in Figure 5B and observe c-TPP with the larger agglomerates by 50 nm. The complexed agglomerates increase with illumination time when with the Rh6G DI water solution. An increased agglomeration response is observed at the higher MWCNT concentrations over the illumination window, and notably, a-TPP complexes offer the highest gradient of any sample. Further DLS data is given in Supporting Information Figures S28–S30.

The photodegradation of Rh6G occurs via N-demethylation and again requires reactive oxygen species as provided following the photoexcitation of organic photocatalysts and the carboxylic functionalized MWCNT—as well as the DI water solvent environment.^48^ Following the formation of a highly reactive hydroxyl radical OH^•^ under photoillumination, carboxylation and dealkylation processes cleave the rhodamine molecules interacting with the radicals to create photodegraded rhodamine products and additional organic intermediate byproducts.^48^

Due to the difference in degradation mechanism between the two rhodamine dyes, it may suggest that the methyl intermediates produced upon photodegradation of Rh6G will adversely affect the c-TPP–*o*-MWCNT complexes to account for the difference in performance between the porphyrins and the carboxylic TPP across the two dyes. Additionally, the lack of a carboxylic group on Rh6G will lead to a lower strength of rhodamine–CNT interaction. It is thought that photocleaving of the porphyrin fluorophore may be more prevalent in the c-TPP–*o*-MWCNT samples under the photocatalysis conditions, although suppressed in aqueous environments.^49^

In summary, efficient electron transfer between the TPP and the *o*-MWCNTs allows for the creation of reactive oxygen species that contribute to the degradation of rhodamine samples. The functional groups attached to the A3B–porphyrin moieties result in a vastly different degradation response due to bonding affinity to the *o*-MWCNT and aggregations of the photocatalyst over the photoillumination period. We also propose the effects of intermediate byproducts following the photodegradation of Rh6G to degrade the performance of the c-TPP–*o*-MWNCT complex catalyst.

## Conclusions

Two A3B porphyrins were adsorbed onto an *o*-MWCNT surface and used to photodegrade both RhB and Rh6G via N-deethylation and N-demethylation, respectively. The use of *o*-MWCNTs allowed for a greater binding affinity of the TPPs to the CNT surface as well as producing the solubility in water needed for use in wastewater environments. The porphyrins chosen were also soluble in water at the concentrations used. Spectroscopic measurements revealed efficient electron transfer between photoexcited porphyrins and the *o*-MWCNTs that could be utilized to create reactive oxygen species that react with rhodamine to give photodegradable products and reduce the toxicity of the sample. Both a-TPP and c-TPP–*o*-MWCNT complexes offered enhanced photocatalytic performance in comparison to the references of rhodamine and the individual TPP or CNT components. However, the decomposition of porphyrin and the formation of larger CNT agglomerates—an effect that was more severe at higher CNT concentrations—over the length of the photoillumination led to a decrease in photocatalytic activity over the timescales of the measurements. We believe that optimal concentrations could be found for long-lasting, recoverable, photodegradation of rhodamine using porphyrin-functionalized CNT complexes at an acceptable efficiency. Additionally, the use of metallated porphyrins may slow down charge recombination and allow for increased photocatalytic activity and recovery of post-degradation catalyst conditions.^50^

## Supporting Information Available

Raw absorption data and the suppressed triplet state (further sample synthesis concerns can be provided following reasonable request) (PDF)
