# Supplementary material for: Porphyrin–Nanocarbon Complexes to Control the Photodegradation of Rhodamine
Source: ACS Omega. 2022 Nov 1;7(45):41304–13. doi: 10.1021/acsomega.2c05065 (PMC9670295; doi:10.1021/acsomega.2c05065)
Supplement: Supplementary file 1 — ao2c05065_si_001.pdf [file ao2c05065_si_001.pdf]

Supplementary Information:

Porphyrin-Nanocarbon Complexes to Control the Photodegradation of Rhodamine

Michael George Spencer,†,‡ Marco Sacchi,†,¶ Jeremy Allam,‡ and S. Ravi P. Silva*,‡
†Quantum Biology Doctoral Training Centre
‡Advanced Technology Institute
¶Department of Chemistry
E-mail of corresponding author: s.silva@surrey.ac.uk

MWCNT Absorption Spectra

Repeat Measurements

Triplet States a-TPP

Porphyrin Decomposition Data – Rhodamine B

1) a-TPP Rhodamine B
2) c-TPP Rhodamine B
3) a-TPP CNT 5μg/ml Rhodamine B
4) a-TPP 40μg/ml Rhodamine B
5) c-TPP 5μg/ml Rhodamine B
6) c-TPP 40μg/ml Rhodamine B

Porphyrin Decomposition Data – Rhodamine 6G

1) a-TPP Rhodamine 6G
2) c-TPP Rhodamine 6G
3) a-TPP CNT 5μg/ml Rhodamine 6G
4) a-TPP 40μg/ml Rhodamine 6G
5) c-TPP 5μg/ml Rhodamine 6G
6) c-TPP 40μg/ml Rhodamine 6G

DLS Agglomeration Data

Solar Simulator Response

MWCNT Absorption Spectra

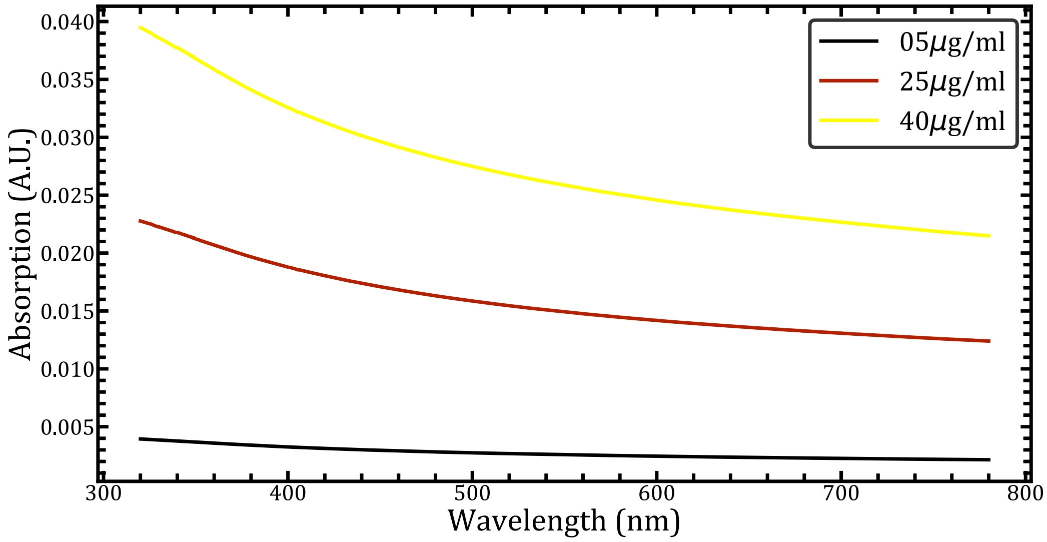

Figure S1: Absorption spectra of o-MWCNTs in DMF at the 3 concentrations of interest. A clear increase in absorption at the high energy end is observed, and a general increase in the background absorption levels throughout.

Repeat Measurements

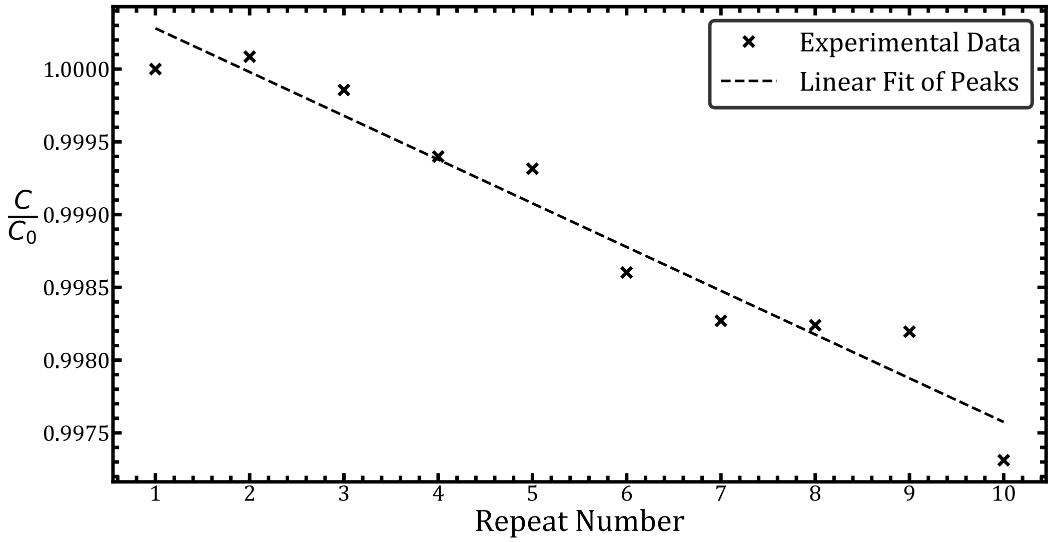

Figure S2: The slight photodegradation of a sample under repeated absorption measurements. The fraction of degradation is predictable and small, as well as behaving similarly for all sample types so will be considered negligible.

Triplet States a-TPP

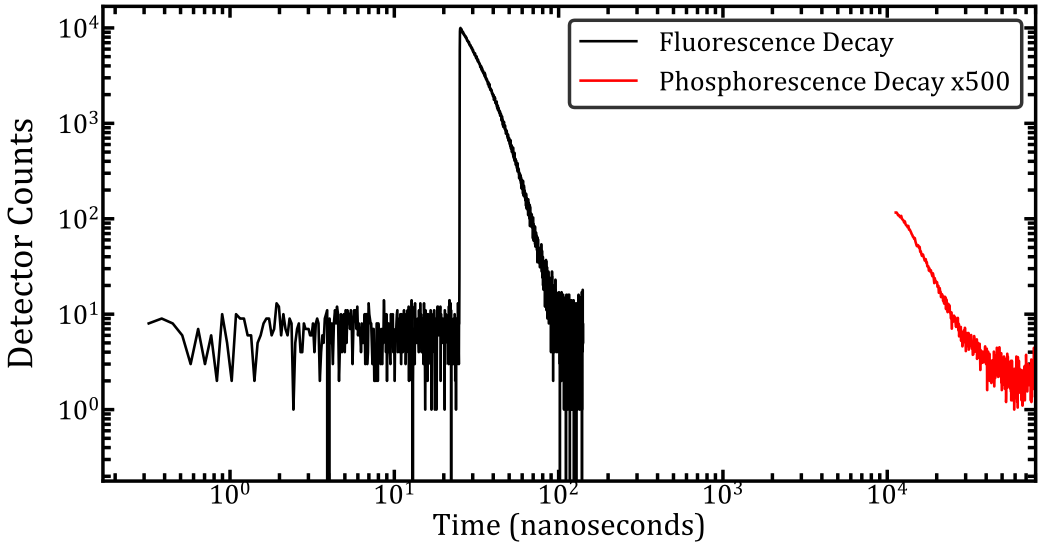

Figure S3: A time-resolved fluorescence trace of a-TPP CNT complexes showing a slow 10us decay indicating a suppressed triplet state when compared to the singlet detector count.

Porphyrin Decomposition Data – Rhodamine B

a-TPP Rhodamine B

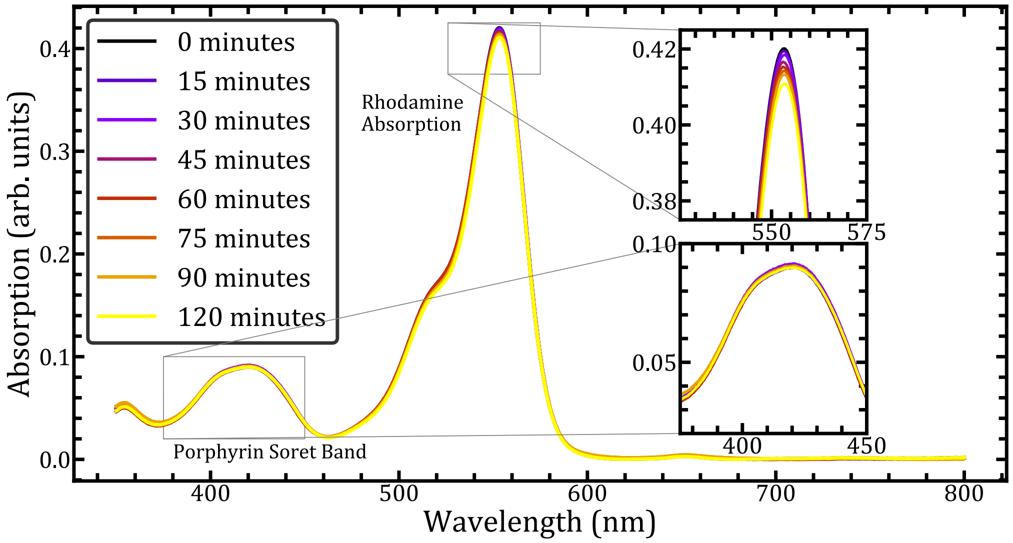

Figure S4: Negligible decrease in Soret band response over course of illumination indicates little change in porphyrin level for the a-TPP rhodamine B samples.

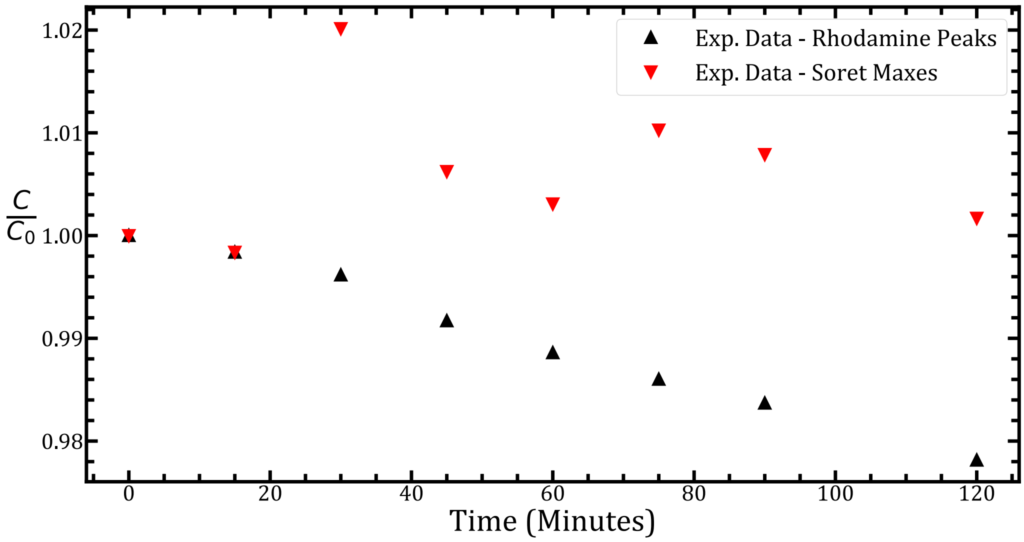

Figure S5: Normalised peak height of Soret band response over course of illumination indicates little change in porphyrin level for the a-TPP rhodamine B samples.

c-TPP Rhodamine B

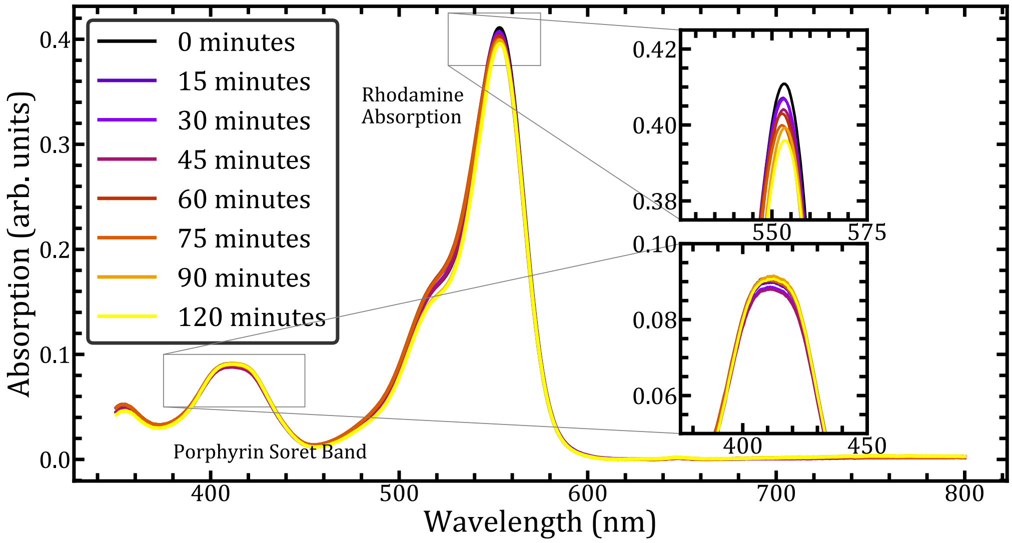

Figure S6: Negligible decrease in Soret band response over course of illumination indicates little change in porphyrin level for the c-TPP rhodamine B samples.

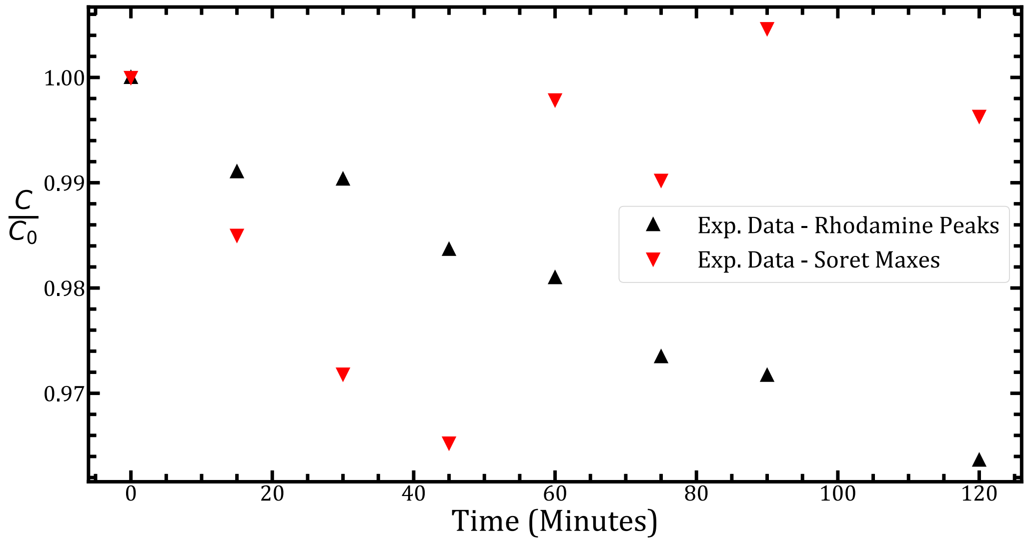

Figure S7: Normalised peak height of Soret band response over course of illumination indicates little change in porphyrin level for the c-TPP rhodamine B samples.

a-TPP CNT05 Rhodamine B

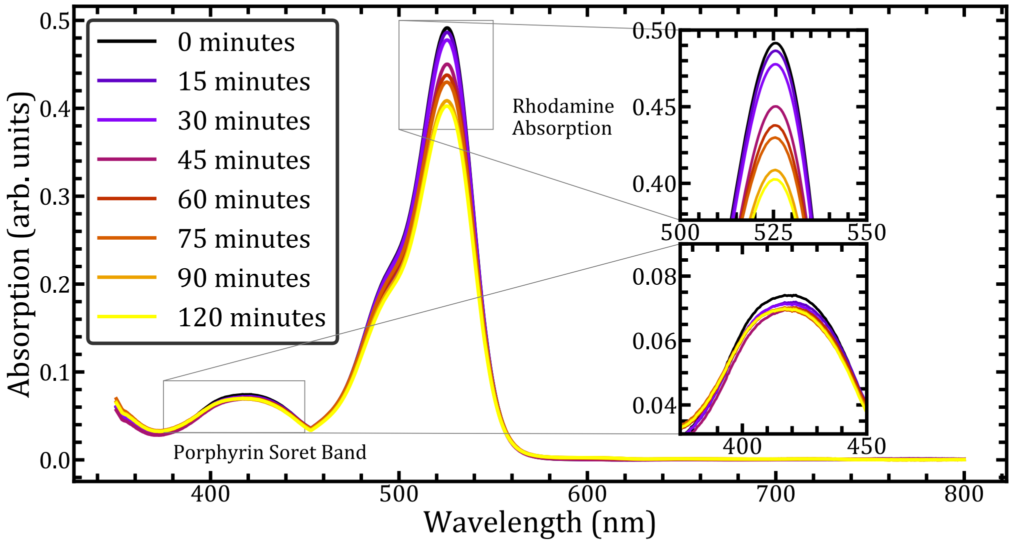

Figure S8: Small decrease in Soret band response over course of illumination for the a-TPP o-MWCNT at 5μg/ml rhodamine B samples.

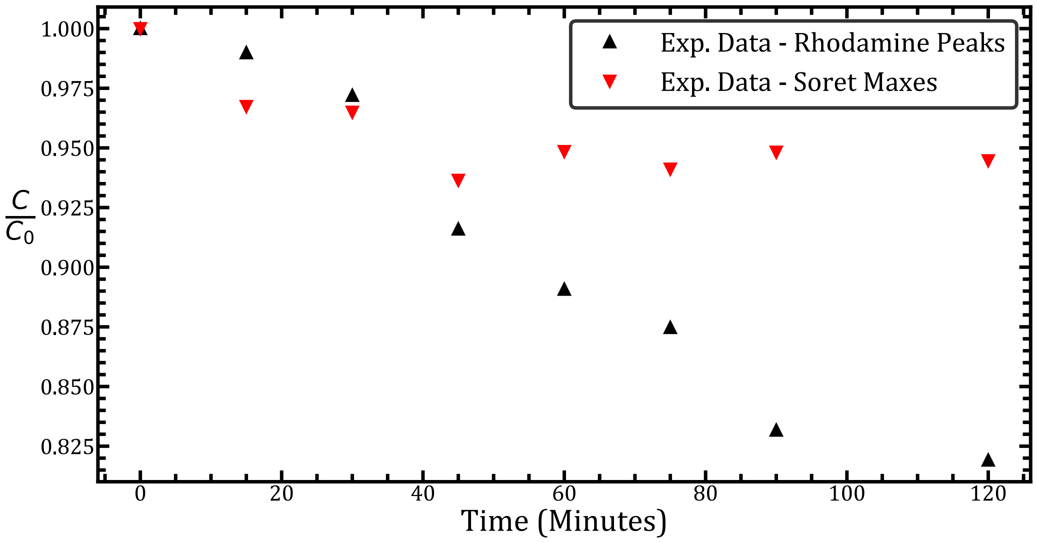

Figure S9: Normalised peak height of Soret band response over course of illumination for the a-TPP o-MWCNT at 5µg/ml rhodamine B samples. A plateau in decomposition occurs beyond 45 minutes.

a-TPP CNT40 Rhodamine B

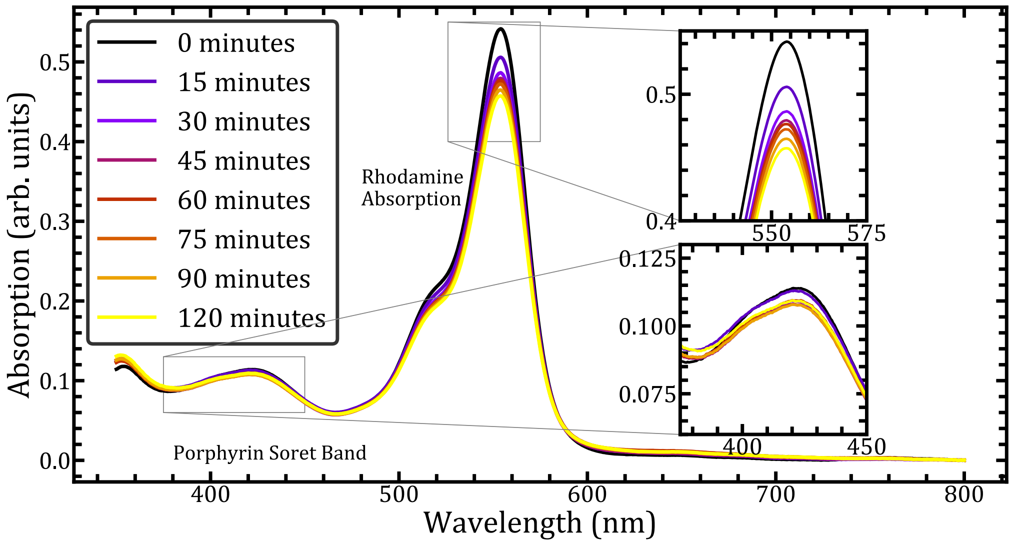

Figure S10: Small decrease in Soret band response over course of illumination for the a-TPP o-MWCNT at 40µg/ml rhodamine B samples.

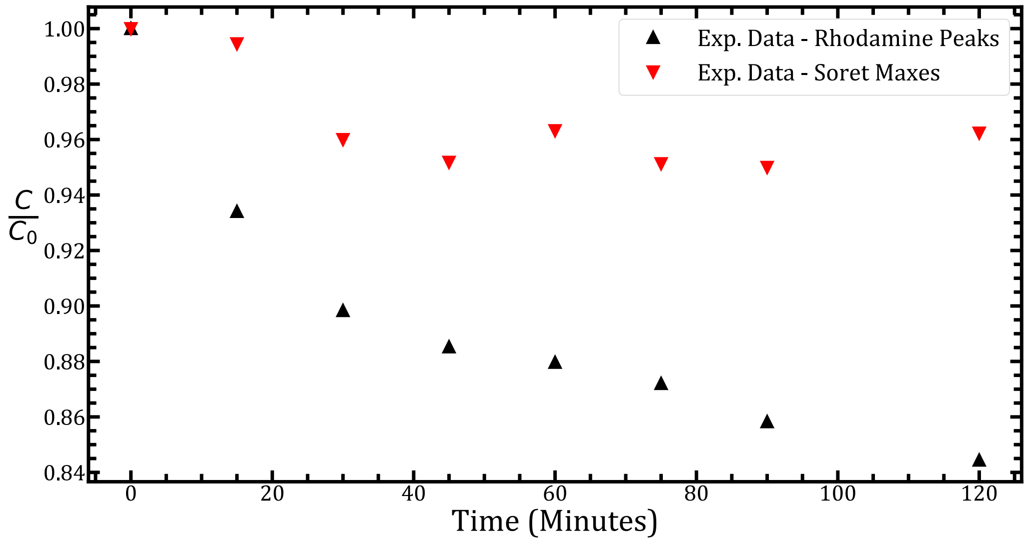

Figure S11: Normalised peak height of Soret band response over course of illumination for the a-TPP o-MWCNT at 40µg/ml rhodamine B samples. A plateau in decomposition occurs beyond 45 minutes.

c-TPP CNT05 Rhodamine B

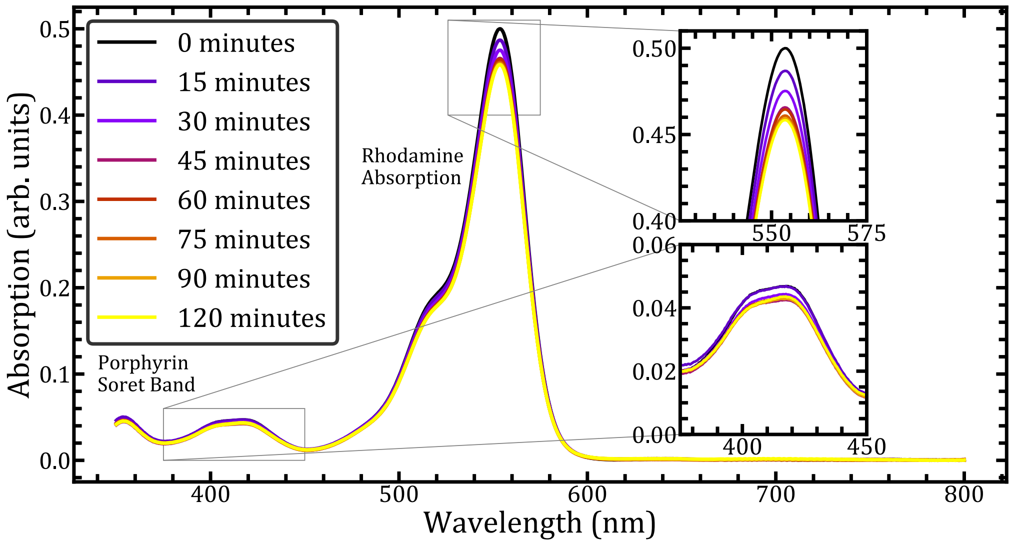

Figure S12: Small decrease in Soret band response over course of illumination for the c-TPP o-MWCNT at 5µg/ml rhodamine B samples.

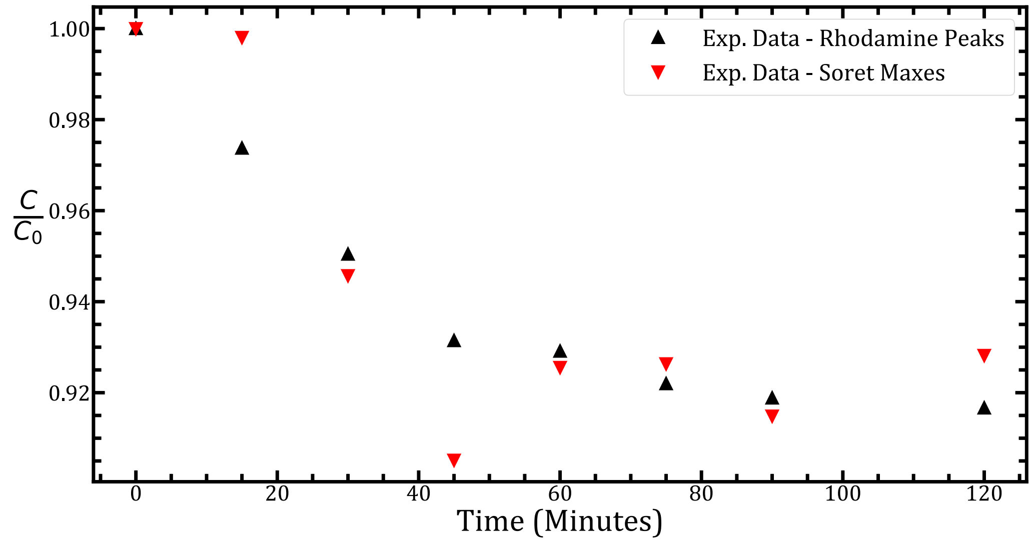

Figure S13: Normalised peak height of Soret band response over course of illumination for the c-TPP o-MWCNT at 5μg/ml rhodamine B samples. A plateau in decomposition again occurs beyond 45 minutes.

c-TPP CNT40 Rhodamine B

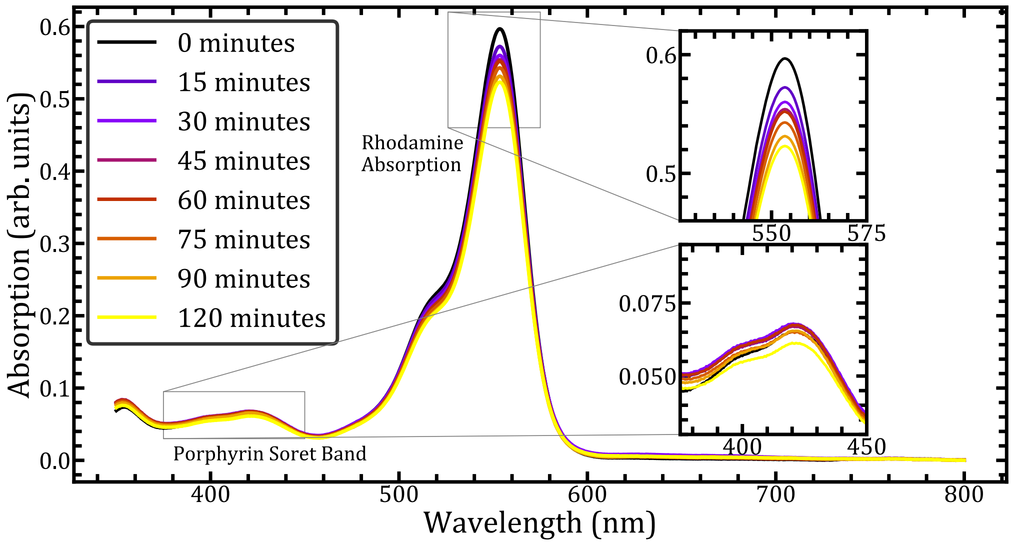

Figure S14: Small decrease in Soret band response over course of illumination for the c-TPP o-MWCNT at 40μg/ml rhodamine B samples. Porphyrin Soret band character displays an unusual shape.

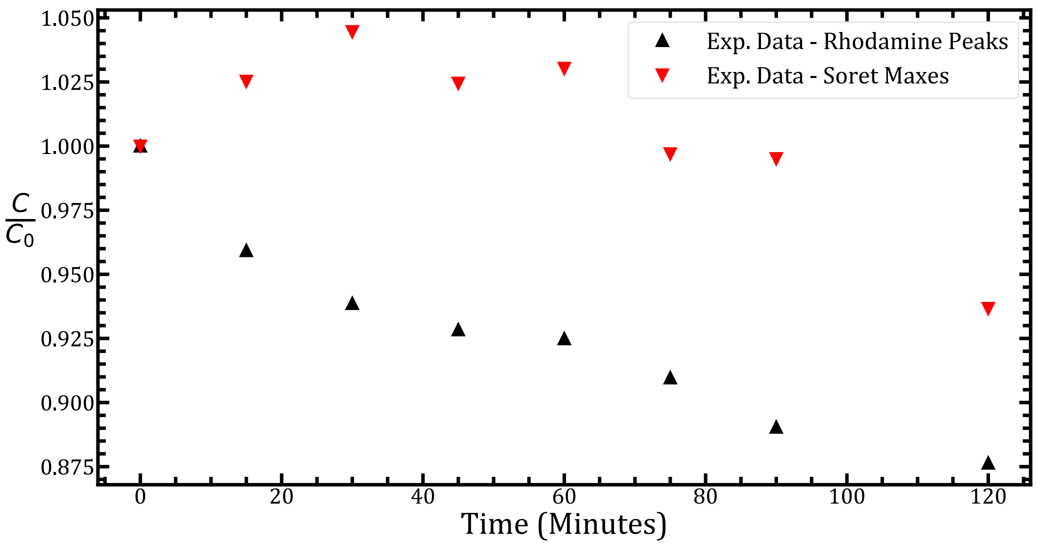

Figure S15: Normalised peak height of Soret band response over course of illumination for the c-TPP o-MWCNT at 40μg/ml rhodamine B samples. A slow decrease, and no plateauing in observed.

Porphyrin Decomposition Data – Rhodamine 6G

a-TPP Rhodamine 6G

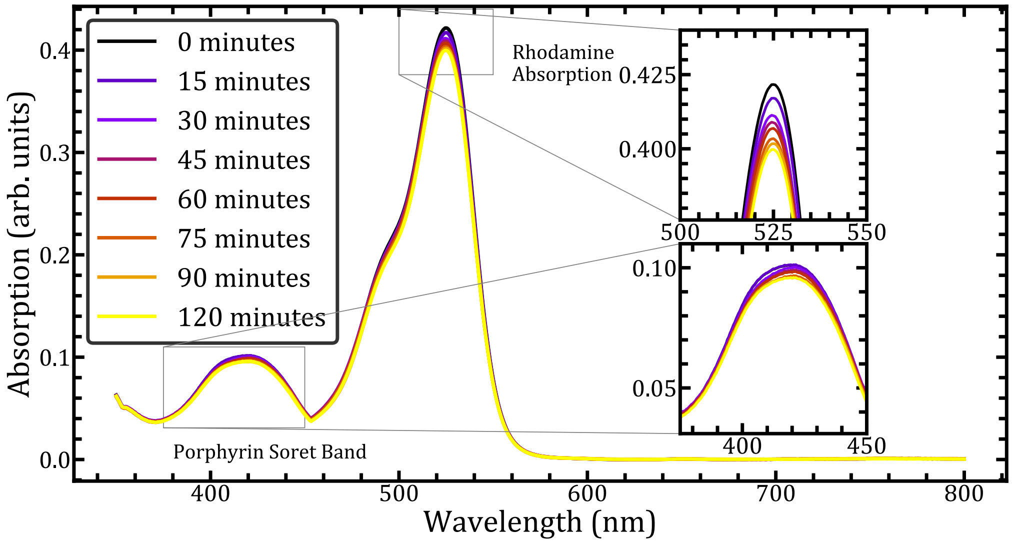

Figure S16: Negligible decrease in Soret band response over course of illumination indicates little change in porphyrin level for the a-TPP rhodamine 6G samples.

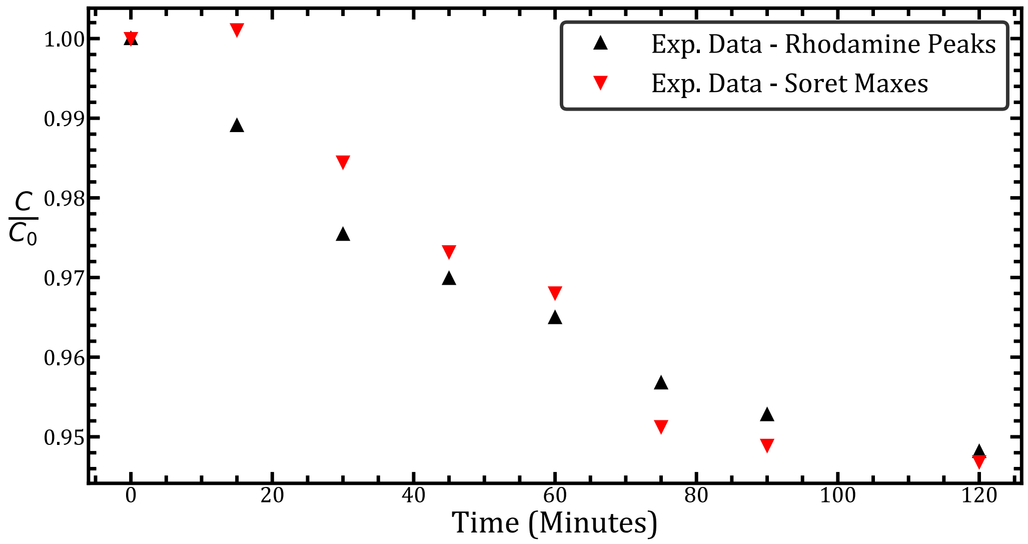

Figure S17: Normalised peak height of Soret band response over course of illumination displays a very linear decrease in porphyrin content to match that of the rhodamine 6G degradation due to a-TPP.

c-TPP Rhodamine 6G

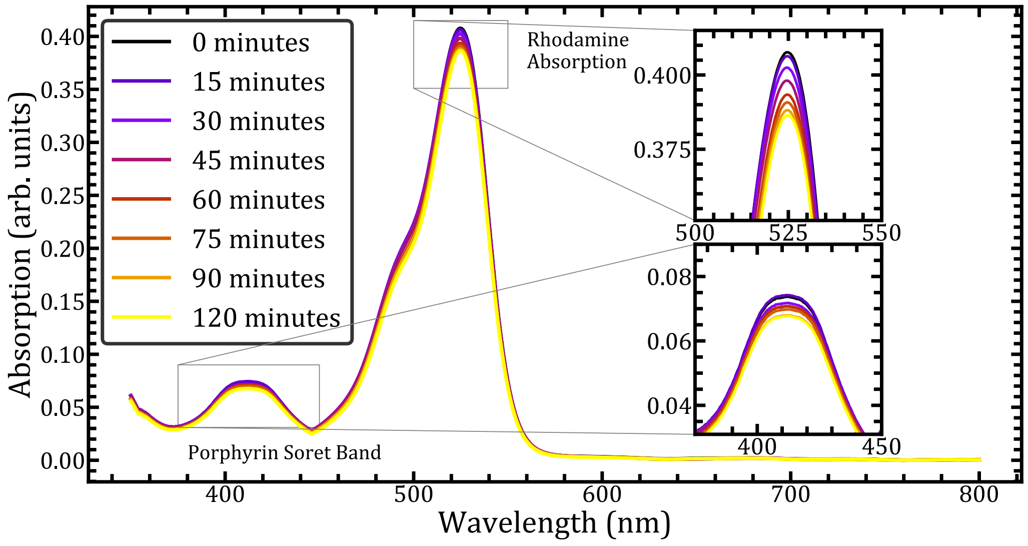

Figure S18: Negligible decrease in Soret band response over course of illumination indicates little change in porphyrin level for the c-TPP rhodamine 6G samples.

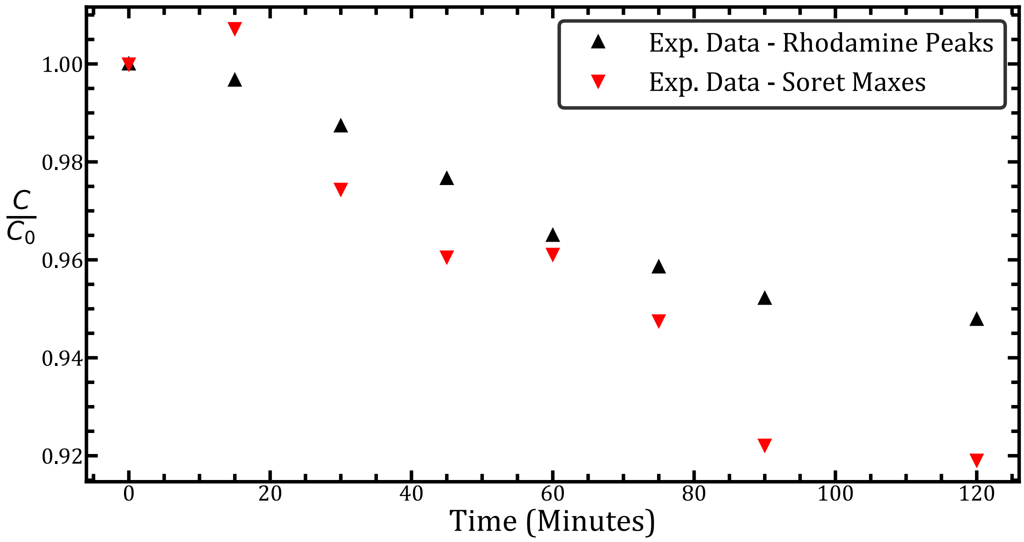

Figure S19: Normalised peak height of Soret band response over course of illumination displays a near linear decrease in porphyrin content to match that of the rhodamine 6G degradation due to c-TPP.

a-TPP CNT05 Rhodamine 6G

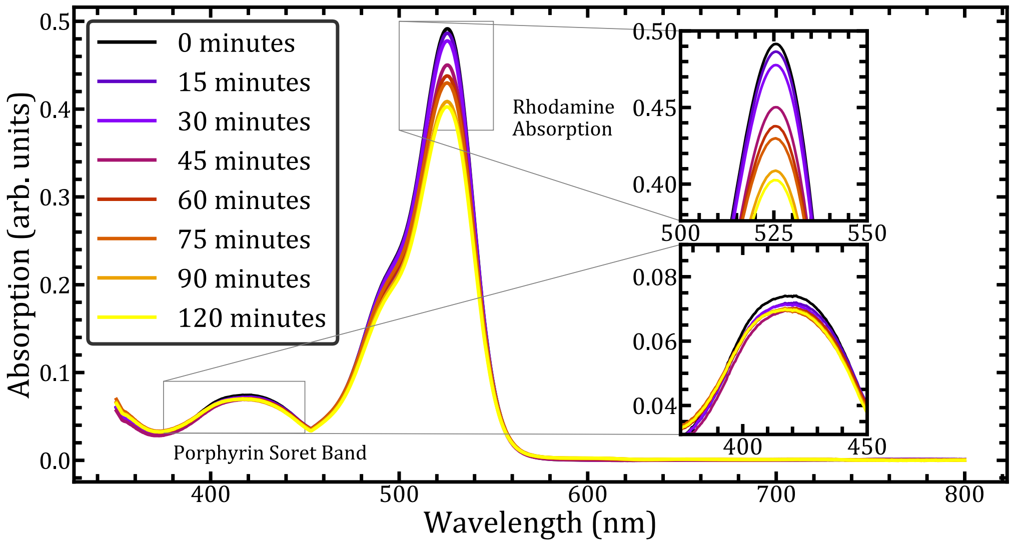

Figure S20: Negligible decrease in Soret band response over course of illumination indicates little change in porphyrin level for the a-TPP o-MWCNT at 5μg/ml complexed samples with rhodamine 6G.

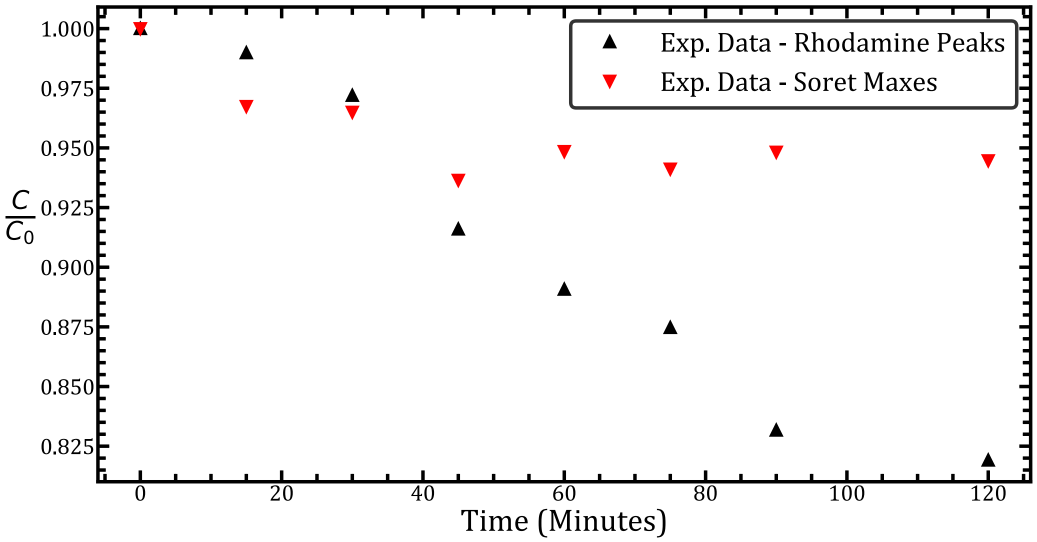

Figure S21: Normalised peak height of Soret band response over course of illumination for the a-TPP o-MWCNT at 5µg/ml rhodamine B samples. A plateau in decomposition again occurs beyond 45 minutes.

a-TPP CNT40 Rhodamine 6G

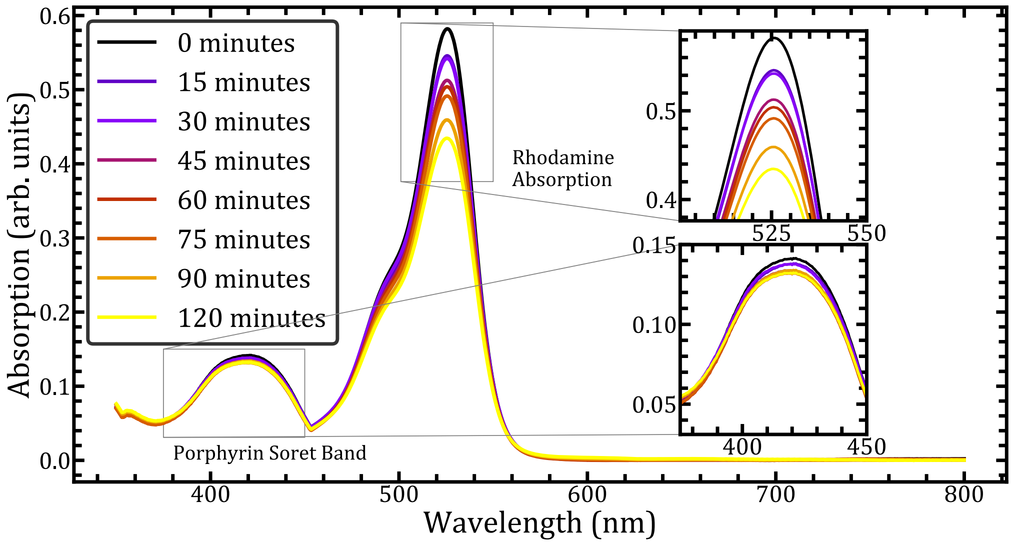

Figure S22: Negligible decrease in Soret band response over course of illumination indicates little change in porphyrin level for the a-TPP o-MWCNT at 40µg/ml complexed samples with rhodamine 6G.

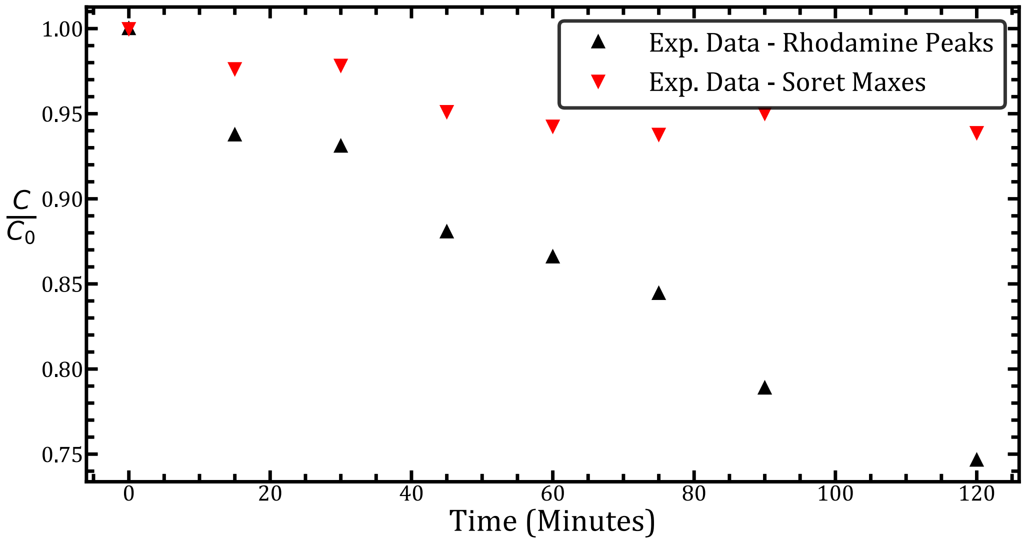

Figure S23: Normalised peak height of Soret band response over course of illumination for the a-TPP o-MWCNT at 40µg/ml rhodamine B samples. A plateau in decomposition again occurs beyond 45 minutes.

c-TPP CNT05 Rhodamine 6G

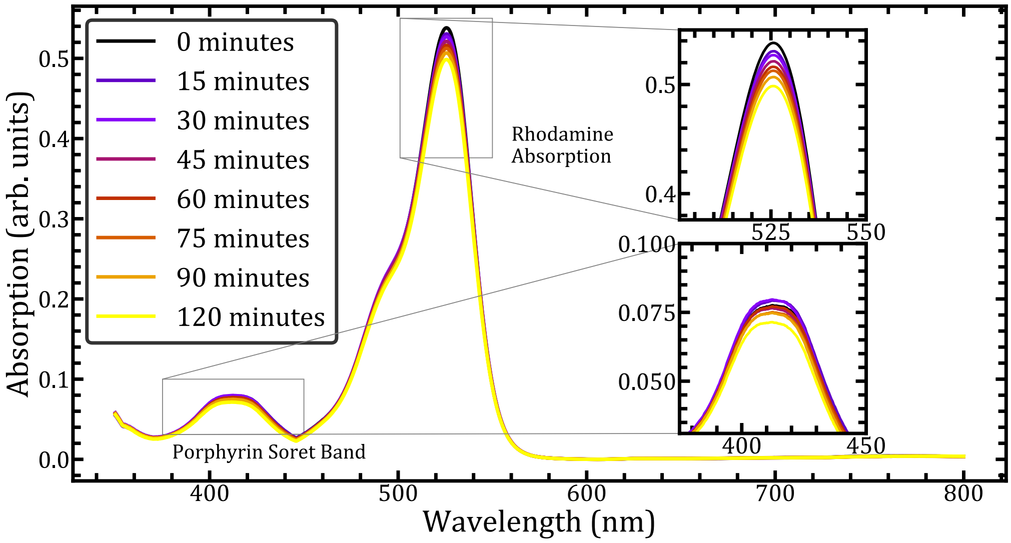

Figure S24: Negligible decrease in Soret band response over course of illumination indicates little change in porphyrin level for the c-TPP o-MWCNT at 5µg/ml complexed samples with rhodamine 6G.

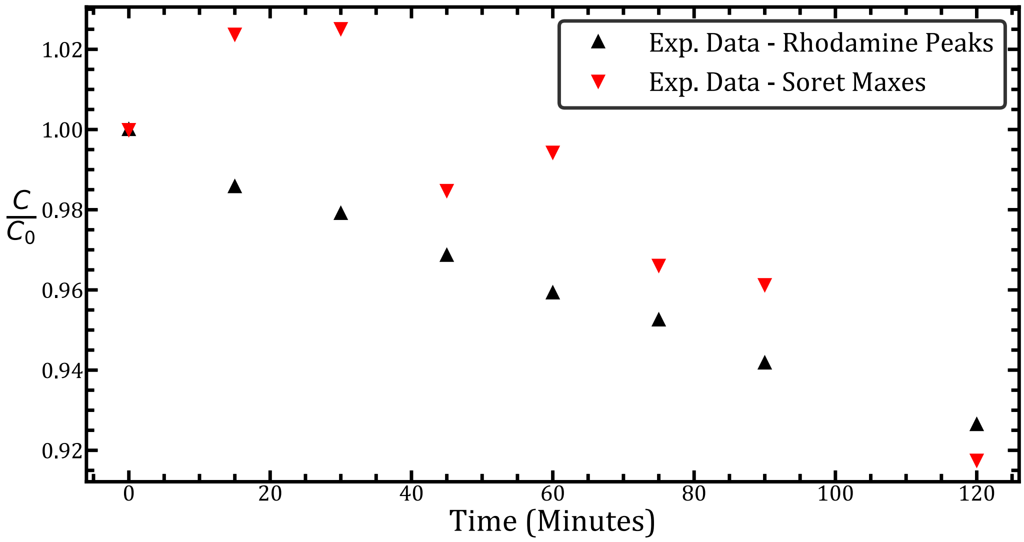

Figure S25: Normalised peak height of Soret band response over course of illumination for the c-TPP o-MWCNT at 5µg/ml rhodamine B samples.

c-TPP CNT40 Rhodamine 6G

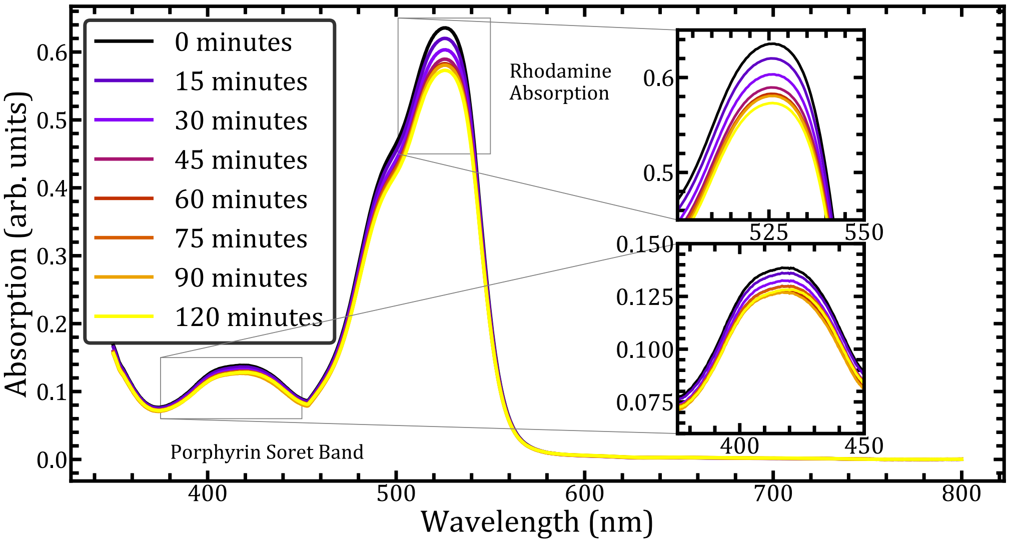

Figure S26: Negligible decrease in Soret band response over course of illumination indicates little change in porphyrin level for the c-TPP o-MWCNT at 40µg/ml complexed samples with rhodamine 6G.

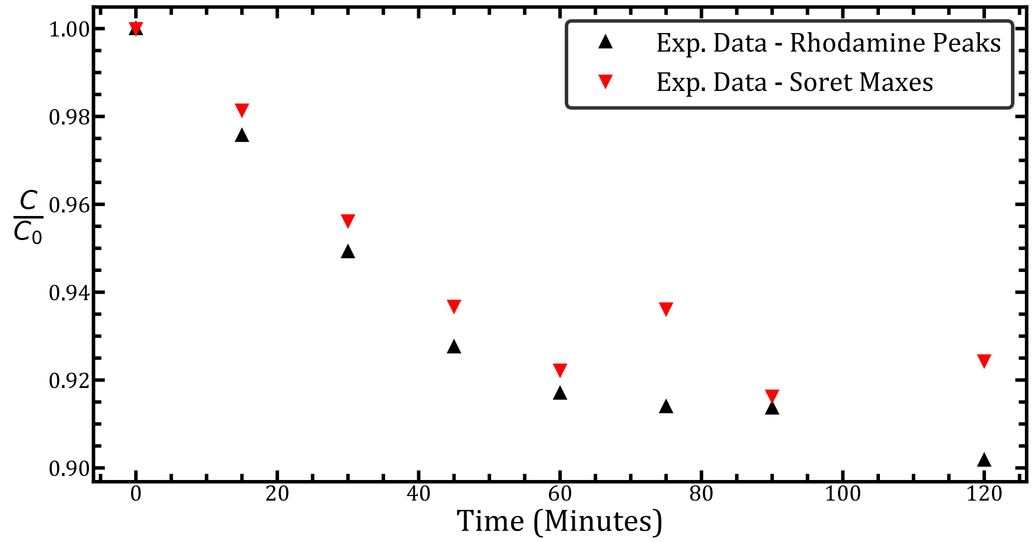

Figure S27: Normalised peak height of Soret band response over course of illumination for the c-TPP o-MWCNT at 5µg/ml rhodamine B samples. A plateau in decomposition again occurs beyond 60 minutes.

DLS Agglomeration Data

Changes to the agglomerate and aggregated structure of our samples in solution were assessed using dynamic light scattering (DLS) techniques to assess particle size. The rhodamines in solution formed aggregates in the 15-30nm range in DI water suggesting the presence of rhodamine-rhodamine linking due to the high concentrations (4mM) of rhodamine B and rhodamine 6G used in experiments. For the rhodamine reference solutions, we observe an increase - near doubling - of particle size over the course of the experiment indicating photoinduced aggregation.

The addition of a-TPP and c-TPP porphyrins to the rhodamine solutions each bring about further aggregation of the components, albeit split between two sizes of different magnitudes. Roughly 30% of a-TPP RhB and Rh6G aggregates ranged from 20-40nm in diameter whilst 70% were in the 100-250 nm range as seen in Figures. 25 and 26. The formation of the agglomerates indicate porphyrin-rhodamine linking. Some porphyrin agglomeration in DI water is noted, but not to the extent seen in the composite solutions. A similar story is found in c-TPP -- RhB solutions, with 20-30% of agglomerates forming in the region of 30-60nm and 70-80% in the region of 150 - 300nm range indicating again porphyrin-rhodamine interaction and slightly larger structures that a-TPP.

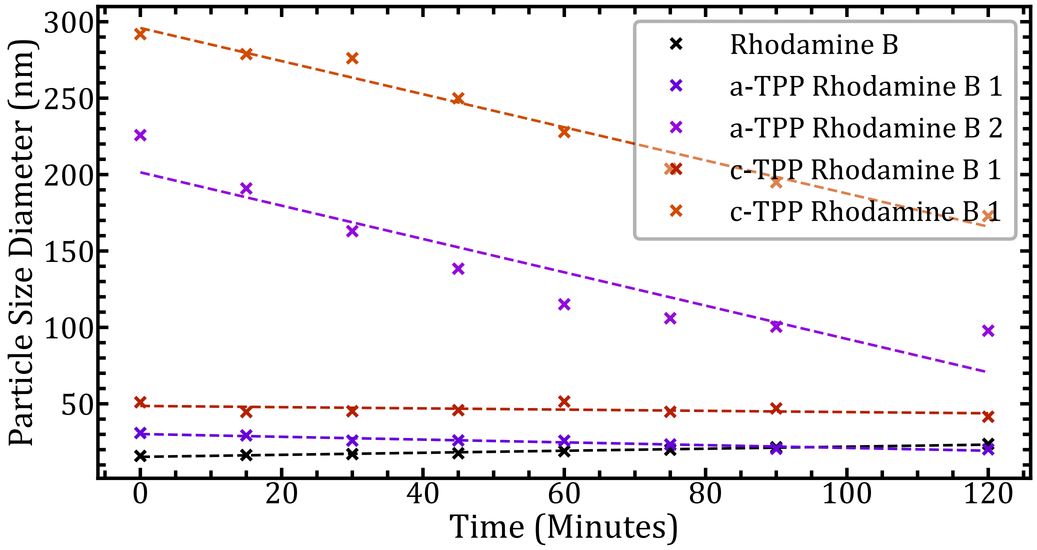

Figure S28: DLS data indicating particle size makeup of rhodamine B reference solutions, and rhodamine B solutions with added a-TPP and c-TPPs.

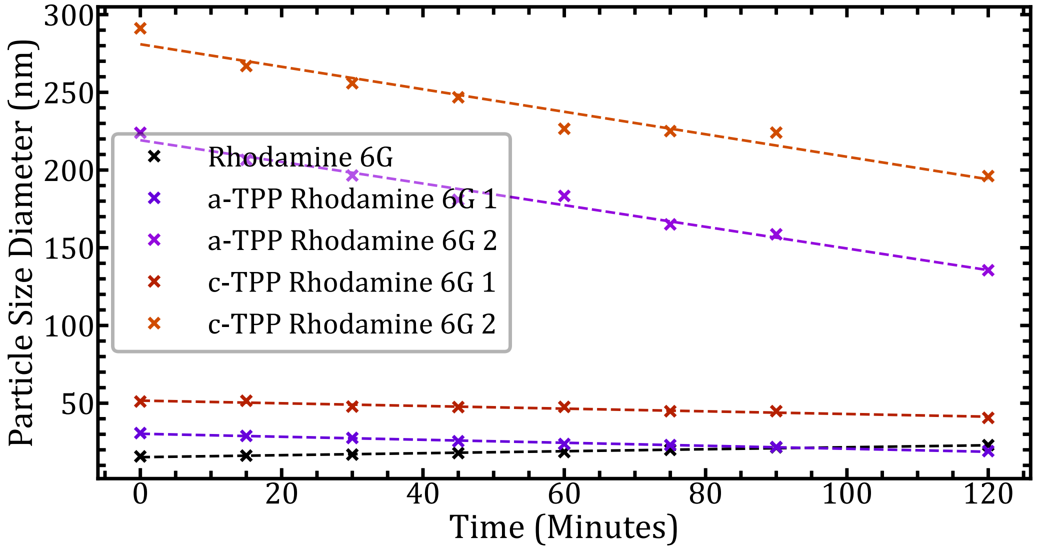

Figure S29: DLS data indicating particle size makeup of rhodamine 6G reference solutions, and rhodamine 6G solutions with added a-TPP and c-TPPs.

The addition of CNTs to a base rhodamine solutions offers split data also, with 50% of agglomerates in the 40-80nm range, and 50% in the 200-400nm range as seen in Figure. 27. The higher concentrations of CNT result in the larger agglomerates being formed as expected. π-π - stacking and cation- π - stacking are expected to be present here and would

account for the interaction between the rhodamines and the acid-functionalised MWCNTs.

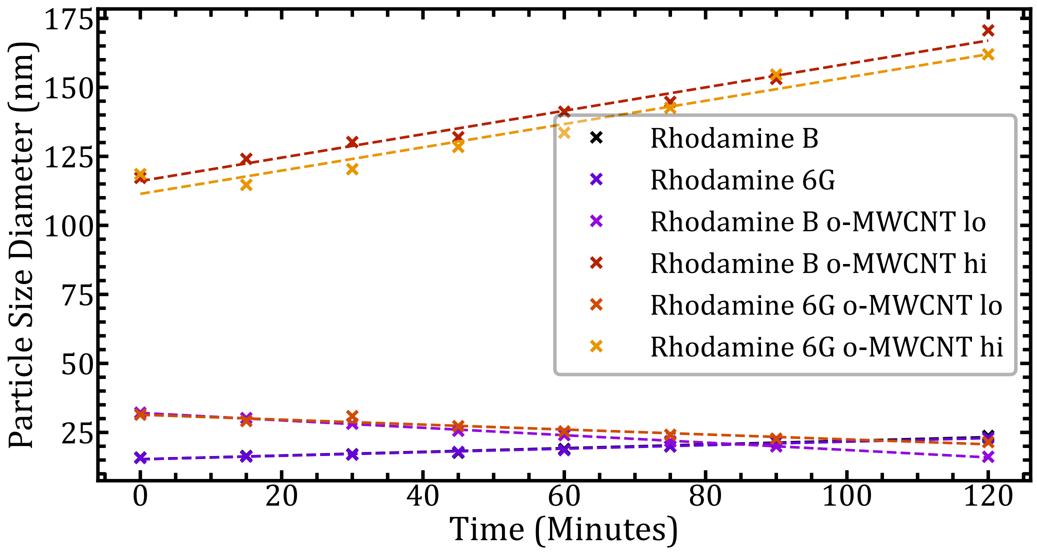

Figure S30: DLS data indicating particle size makeup of rhodamine reference solutions, and rhodamine solutions with added o-MWCNTs at a concentration 40µg/ml.

For the full complexes in rhodamine-porphyrin -- o-MWCNT solutions we see a removal of the split large particle size character entirely within the DLS data. Instead, some scattering of isolated pigments in the 1-5nm range are observed at a small amplitude, whilst a larger distribution of particles in the 120-400nm range are observed. It is worth noting that the stacking and agglomeration characteristics of a raw MWCNT and porphyrin decorated MWCNTs are expected to be different, accounting for this change. Interestingly, if unattached porphyrins were expected in high numbers we would expect possibly aggregates in the 30nm range and this is not observed. We therefore conclude that even highly decomposed photocatalysts are not cleaving porphyrins from the CNT surface but rather forming a rate change via a larger structure being formed and leading to surface area - reaction rate arguments.

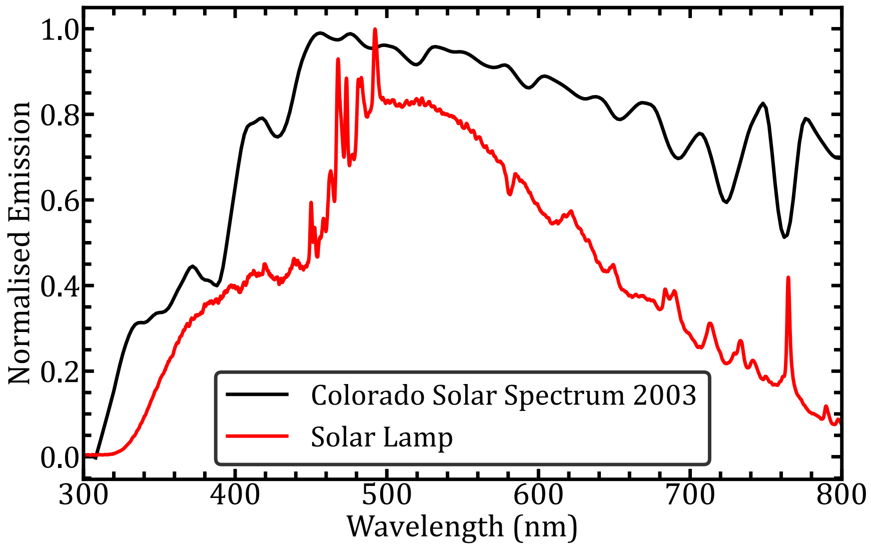

Figure S31: Spectral data for class-b solar simulator used for photodegradation illumination as compared to a standard solar response (Colorado Midday Standard 2003).
